# Inflammation-related microRNA expression level in the bovine milk is affected by mastitis

**DOI:** 10.1371/journal.pone.0177182

**Published:** 2017-05-17

**Authors:** Yu-Chang Lai, Takuro Fujikawa, Tadashi Maemura, Takaaki Ando, Go Kitahara, Yasuyuki Endo, Osamu Yamato, Masateru Koiwa, Chikara Kubota, Naoki Miura

**Affiliations:** 1 Veterinary Teaching Hospital, Joint Faculty of Veterinary Medicine, Kagoshima University, Kagoshima, Japan; 2 The United Graduate School of Veterinary Science, Yamaguchi University, Yamaguchi, Japan; 3 Laboratory of Veterinary Theriogenology, Joint Faculty of Veterinary Medicine, Kagoshima University, Kagoshima, Japan; 4 Department of Veterinary Sciences, Faculty of Agriculture, University of Miyazaki, Miyazaki, Japan; 5 Laboratory of Small Animal Internal Medicine, Joint Faculty of Veterinary Medicine, Kagoshima University, Kagoshima, Japan; 6 Laboratory of Veterinary Clinical Pathology, Joint Faculty of Veterinary Medicine, Kagoshima University, Kagoshima, Japan; 7 School of Veterinary Medicine, Rakuno Gakuen University, Ebetsu, Hokkaido, Japan; Gustave Roussy, FRANCE

## Abstract

MicroRNA (miRNA) in tissue and liquid samples have been shown to be associated with many diseases including inflammation. We aimed to identify inflammation-related miRNA expression level in the bovine mastitis milk. Expression level of inflammation-related miRNA in milk from mastitis-affected and normal cows was analyzed using qPCR. We found that expression level of miR-21, miR-146a, miR-155, miR-222, and miR-383 was significantly upregulated in California mastitis test positive (CMT+) milk. We further analyzed these miRNA using a chip-based QuantStudio Digital PCR System. The digital PCR results correlated with those of qPCR, demonstrating upregulation of miR-21, miR-146a, miR-155, miR-222, and miR-383 in CMT+ milk. In conclusion, we identified miRNA that are upregulated in CMT+ milk. These miRNA exhibited sensitivity and specificity greater than 80% for differentiating between CMT+ milk and normal milk. Our findings suggest that inflammation-related miRNA expression level in the bovine milk was affected by mastitis, and miRNA in milk have potential for use as biomarkers of bovine mastitis.

## Introduction

MicroRNA (miRNA) are small noncoding RNA molecules of 18–25 nucleotides. miRNA regulate gene expression at the post-transcriptional level by causing RNA degradation or blocking mRNA translation. miRNA in tissue and liquid samples have been shown to be associated with many diseases including inflammation [1–3]. Thus, miRNA have specific roles in disease and inflammation pathogenesis. miRNA have been investigated as noninvasive biomarkers of various diseases in humans, including cancer, cardiac disease, infection, and inflammatory disease [4, 5]. miRNA have also been identified in the milk of cows [6, 7], pigs [8], humans [9], goats [10], rats [11], and yaks [12]. miRNA in milk are stable and resistant to acidic environments, RNase digestion, incubation at room temperature and multiple freeze/thaw cycles [7, 13–16]. This suggests that miRNA in milk could potentially be used as biomarkers or for quality control [6].

Bovine mastitis is an inflammatory disease with clinical and subclinical types. Early detection and treatment facilitates early recovery without significant reduction in reproductive performance and helps to prevent transmission to other cows. Therefore, specific and sensitive biomarkers for early detection of mastitis to allow early treatment need to be identified. The most frequently used diagnostic methods for bovine mastitis are somatic cell counting and bacteriological culturing of milk samples [17]. California Mastitis Test (CMT) is the cowside test to estimate somatic cell count range on dairy farm. CMT reagent disrupt the cells in milk sample and react with the DNA in those cells. After reaction, color changes the reagent and rennet reaction are recorded to estimate somatic cell number range, indicating the severity of the inflammation. The limitations of CMT including the interpretation of the results may vary between testers, and other conditions that influence milk somatic cell count apart from infection such as parity, season and stress [18, 19].

Quantitative polymerase chain reaction (qPCR) is a commonly used and indirect technology for evaluating miRNA expression level that allows only relative quantification. qPCR quantifies the number of cycles required for the fluorescent signal to exceed background fluorescence (the CT value); this value is then normalized to data for internal control genes to allow calculation of the relative expression level of target miRNA. Digital PCR is another, chip-based PCR technology that can quantify absolute miRNA expression level directly by PCR and Poisson statistical analysis of fluorescent signals from positive and negative wells. Digital PCR has greater precision and day-to-day reproducibility than qPCR, and comparable sensitivity [20].

In the in the preliminary study, we select several inflammation related miRNA. miR-21 regulates proinflammatory protein PDCD4 expression level after lipopolysaccharide (LPS) stimulation [21]. miR-26 regulates inflammation through down-regulating IL-6 production [22], and miR-26b participates in the inflammatory response of LPS stimulated bovine alveolar macrophages by enhancing the NF-κB signaling pathway [23]. miR-29b is repressed by NF-κB pathway [24], and miR-29b can repress TNFAIP3, a negative regulator of NF-κB pathway [25]. LPS induced inflammation increases blood levels of miR-122 [26]; serum miR-122 also correlates with mortality in human sepsis patients [27, 28]. miR-125b is down-regulated in bovine CD14+ monocytes stimulated with *Staphylococcus aureus* enterotoxin B [29] and activate the NF-κB pathway by targeting TNFAIP3 [30]. miR-204 mediates vascular inflammation in high fat diet mice [31], and plays a role in the regulation of inflammation process through promoting the expression level of SIRT1 and attenuating of inflammatory factors [32, 33]. miR-205, which expression level is upregulated upon NF-kB activation, reduces COMMD1 expression level. The miR-205-COMMD1-NF-κB axis enhances inflammatory response [34]. miR-222 is involved in the pathogenesis of inflammatory diseases, such as rheumatoid arthritis, atherosclerosis and obesity-related inflammation [35, 36]. The mechanism associate with adhesion and infiltration of inflammatory cells into the endothelial space [37]. miR-383 expression level is upregulated in LPS induced macrophage cell line RAW264.7 [38]. Among these miRNA, miR-26b, miR-29b, miR-122, and miR-205 were differentially expressed in the serum of cow with metritis [39]. We included miR-146a and miR-155 in the second phase of this study. miR-146a and miR-155 are well characterized and first reported inflammation-related miRNA [40]. miR-146a and miR-155 expression level are induced by expose human acute monocytic leukemia cell line THP-1 to LPS [40]. miR-146a expression levels are significantly increased in bovine mammary tissues infected with subclinical, clinical and experimental mastitis [41].

In this study, we used qPCR and QuantStudio 3D Digital PCR to investigate differences in inflammation-related miRNA expression level in three groups of milk samples: milk from normal cows; and milk from California mastitis test-negative (CMT−) and -positive (CMT+) quarters of mastitis-affected cows. We identified that miR-21, miR-146a, miR-155, miR-222, and miR-383 were significantly upregulated in CMT+ milk. These miRNA had sensitivity and specificity greater than 80% for differentiating between CMT+ milk and milk from normal cows. Our findings suggest that inflammation-related miRNA expression level in the bovine milk was affected by mastitis, and miRNA in milk have potential for use as biomarkers of bovine mastitis.

## Materials and methods

### Milk sample preparation

All of the milk samples were taken from milking Holstein-Friesian cows. The cows were kept in free-stall barn or tie-stall and pasture without grazing systems; milked twice a day. The animals were fed twice daily, and water was available ad libitum. Milk samples (approximately 5–10 ml) were collected and immediately screened in the field using a modified California Mastitis Test (CMT) with a commercial tester ("PL Tester", Nippon Zenyaku Kogyo) as previously described [42]. Cows with no CMT+ result for any quarter were defined as the normal group; cows with a CMT+ result for at least one quarter were defined as the mastitis-affected group. On the basis of the CMT results, each quarter of the mastitis-affected group was defined as CMT− or CMT+ as appropriate. Reducing sampling and quarter bias, the samples were collected from the commercial farms of three different locations in Japan (Kagoshima, Miyazaki and Hiroshima prefecture). The separated single quarter samples and mixed quarter samples were used in normal group; mastitis samples were taken randomly from cranial and caudal, left and right side of cow quarters. Considering the effect of season on the cow and milk, the samples were collected at different time points including winter and summer (S1 and S2 Tables). The samples were stored at 4°C after collection and transported to the laboratory, then centrifuged at 3000 g for 15 min to remove cell debris and fat. The supernatant was recovered and further centrifuged at 15000 × g for 15 min. The milk whey was recovered and stored at −80°C for RNA extraction.

### Total RNA extraction

Total RNA was extracted from 300 μL milk using a mirVana PARIS kit (Thermo Fisher Scientific) according to the manufacturer’s protocol. The concentration of total RNA was too low to be detected with the NanoDrop 2000c (Thermo Fisher Scientific). The quality of RNA was assessed using the Small RNA kit in combination with the 2100 Bioanalyzer System (Agilent)(S1 Fig).

### Quantification of miRNA by qPCR

Equal volumes of RNA (1.25 microliter) were reverse transcribed to cDNA using TaqMan MicroRNA Assays (Thermo Fisher Scientific) according to the manufacturer’s protocol. qPCR was performed using a TaqMan Fast Advanced Master Mix kit and a StepOne Plus Real Time PCR system (Thermo Fisher Scientific). Thermal cycling was conducted according to the manufacturer’s recommended protocol, and all experiments were performed in duplicate. ΔCT was calculated by subtracting the CT values of miR-92a [43] from the CT value of the target miRNA. ΔΔCT was calculated by subtracting the mean target miRNA ΔCT value from the ΔCT value of normal, CMT−, or CMT+ samples. Expression level was determined using the 2^−ΔΔCT^ method. qPCR reactions of undetermined CT were assigned CT = 40. The TaqMan MicroRNA Assays used in this study and their IDs are as follows: miR-21 (ID: 000397), miR-29b (ID: 000413), miR-92a (ID: 000431), miR-122 (ID: 002245), miR-125b (ID: 000449), miR-146a (ID: 005896_mat), miR-155 (ID: 002623), miR-204 (ID: 000508), miR-205 (ID: 000509), miR-222 (ID: 002276), and miR-383 (ID: 000573).

### Quantification of miRNA by digital PCR

Digital PCR was performed using the QuantStudio 3D Digital PCR System (Thermo Fisher Scientific) according to the manufacturer’s protocol. In brief, 3 μL cDNA of miR-146a, miR-155, miR-222, and miR-383 were combined with QuantStudio 3D Digital PCR Master Mix and TaqMan Assay. The cDNA of miR-21 was diluted 1:30 with RNase-free water before being combined with the reagent because miR-21 was highly expressed and exceeded the instrument detection range. The samples were loaded onto chips using QuantStudio 3D Digital PCR Chip Loader (Thermo Fisher Scientific). The manufacturer’s recommended digital PCR thermal cycling protocol was used. After PCR, the fluorescence data from the chips were collected using a QuantStudio 3D Digital PCR Instrument and uploaded to QuantStudio 3D Analysis Suite Cloud Software for further analysis.

### Statistics

Data analysis was performed using GraphPad Prism 6 (GraphPad Software Inc., San Diego, CA). Data were compared using a parametric unpaired t-test, or one-way ANOVA followed by Tukey’s test where appropriate. Differences were considered to be significant at *P* < 0.05. The area under the curve (AUC), cut-off point, sensitivity, and specificity were analyzed by receiver operating characteristic curve. Cut-off points were determined by the Youden index [44]. Correlation analysis was performed using Pearson’s correlation coefficient.

## Results

### Identification of miRNA with altered expression level in milk from mastitis-affected cows and CMT+ milk

**Fig 1:**
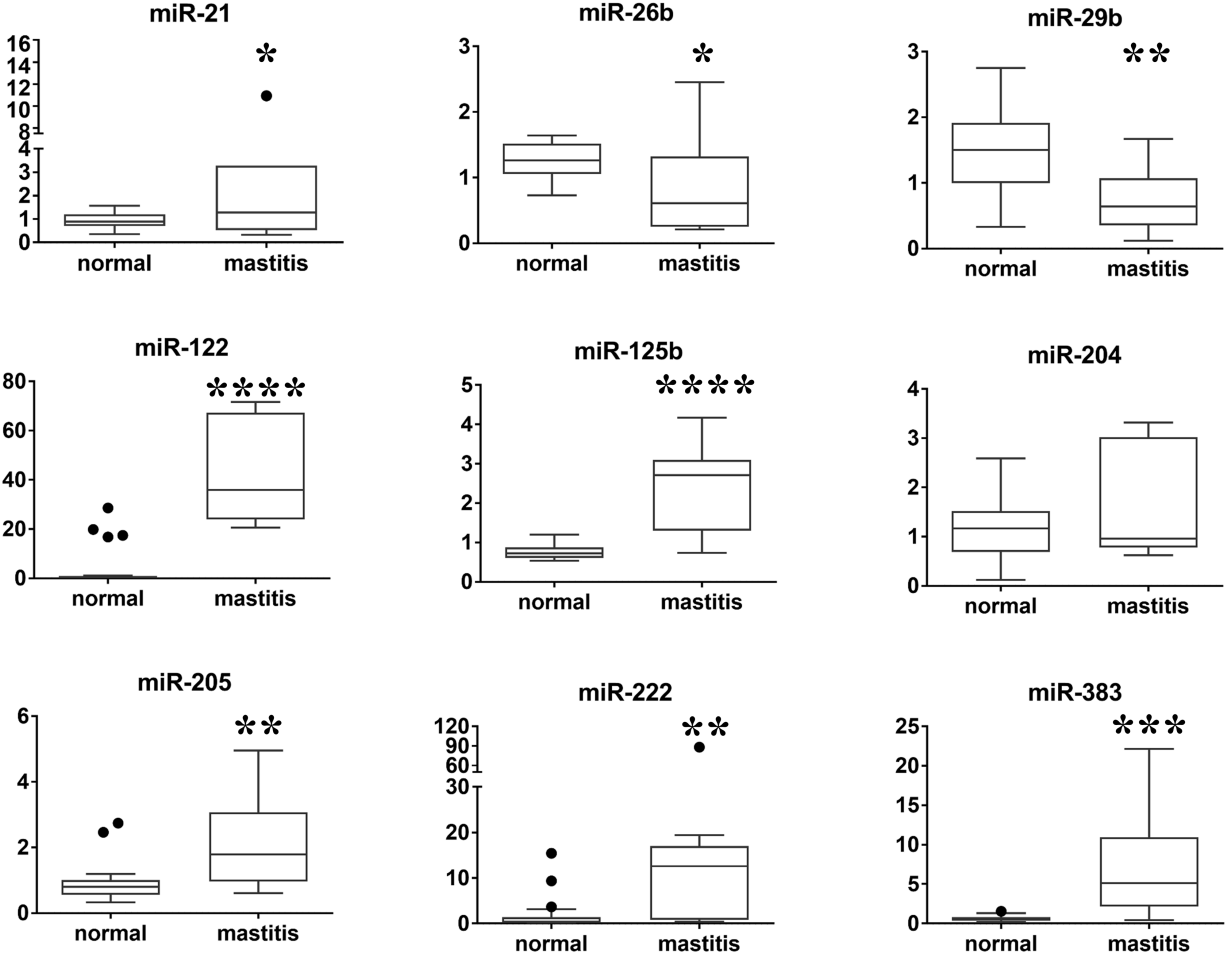
Relative expression levels of nine miRNA in milk from mastitis-affected cows and normal cows using qPCR. Boxes indicate the median, and 25th and 75th quartiles. Whiskers extend from the edge of the box to 1.5 times the interquartile range, and dots indicate data points outside this range. The y-axes represent relative miRNA expression levels in arbitrary units (parametric unpaired t-test, *P < 0.05, **P < 0.01, ***P <0.001, ****P <0.0001).

We included six cows without mastitis (n = 22) and three mastitis-affected cows (n = 9; two mastitis-affected cows (n = 7) for miR-21) in the preliminary study. We analyzed the expression levels of nine inflammation-related miRNA in milk by qPCR and normalized the values obtained to the expression level of miR-92a [43]. Six miRNA (miR-21, miR-122, miR-125b, miR-205, miR-222, and miR-383) were significantly upregulated and two miRNA (miR-26b and miR-29b) were significantly downregulated in milk from mastitis-affected cows, as compared with that from normal cows (Fig 1, S3A and S3B Table). The preliminary study suggests that bovine milk contains inflammation-related miRNA which expression level may affected by mastitis.

**Fig 2:**
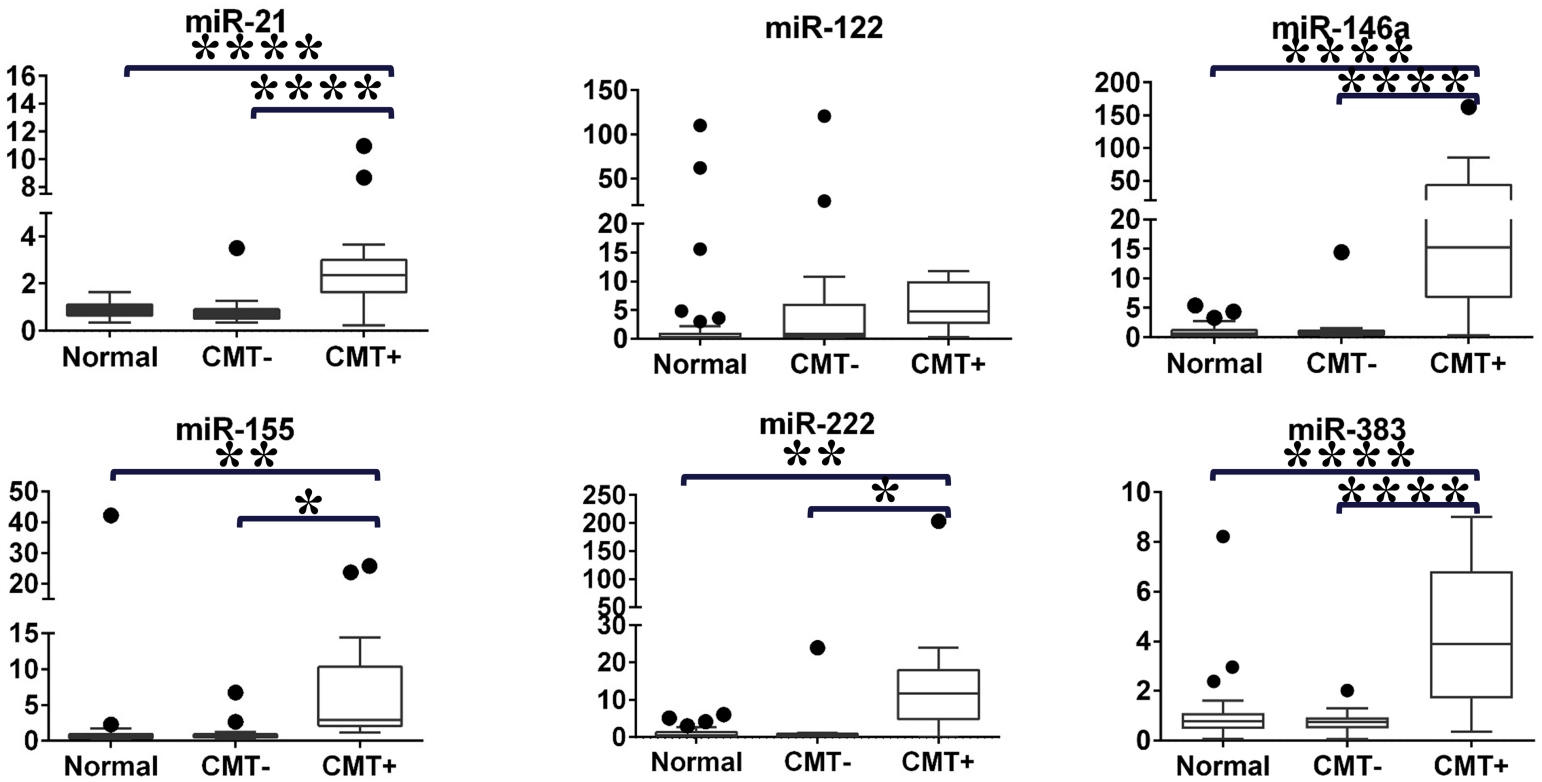
Relative expression levels of six miRNA in milk from normal cows, and from CMT− and CMT+ quarters using qPCR. Boxes indicate the median, and 25th and 75th quartiles. Whiskers extend from the edge of the box to 1.5 times the interquartile range, and dots indicate data points outside this range. The y-axes represent relative miRNA expression levels in arbitrary units (One-way ANOVA followed by Tukey’s test, *P < 0.05, **P < 0.01, ****P <0.0001).

To confirm and further assess the expression level of inflammation-related miRNA, we increased the number of samples and analyzed selected miRNA. We selected miR-21, miR-122, miR-222, and miR-383, which were highly upregulated in milk from mastitis-affected cows in the preliminary study. Additionally, we included miR-146a and miR-155, which are known to be related to inflammation [40], as candidates in the second phase experiment. The second study included milk from 18 normal cows (n = 42) and 14 mastitis-affected cows. Bovine mastitis can be classified as contagious mastitis [45] and environmental mastitis [46]. Contagious mastitis may spread from infected quarters to other quarters or cows. Environmental mastitis is caused by expose to environmental pathogens in the dairy farm environment. To investigate if microRNA expression level in the CMT negative quarters would be implicated by mastitis, we separated the milk from mastitis-affected cows into CMT− (n = 18) and CMT+ (n = 17) groups. The expression levels of five miRNA (miR-21, miR-146a, miR-155, miR-222, and miR-383) were significantly upregulated in the CMT+ group, compared with in the CMT− and normal groups. The expression levels of all miRNA did not differ between the CMT− and normal groups. There was no significant difference in miR-122 levels among the three groups, so this miRNA was excluded from further study (Fig 2 and S4 Table).

### Receiver operating characteristic analysis

**Fig 3:**
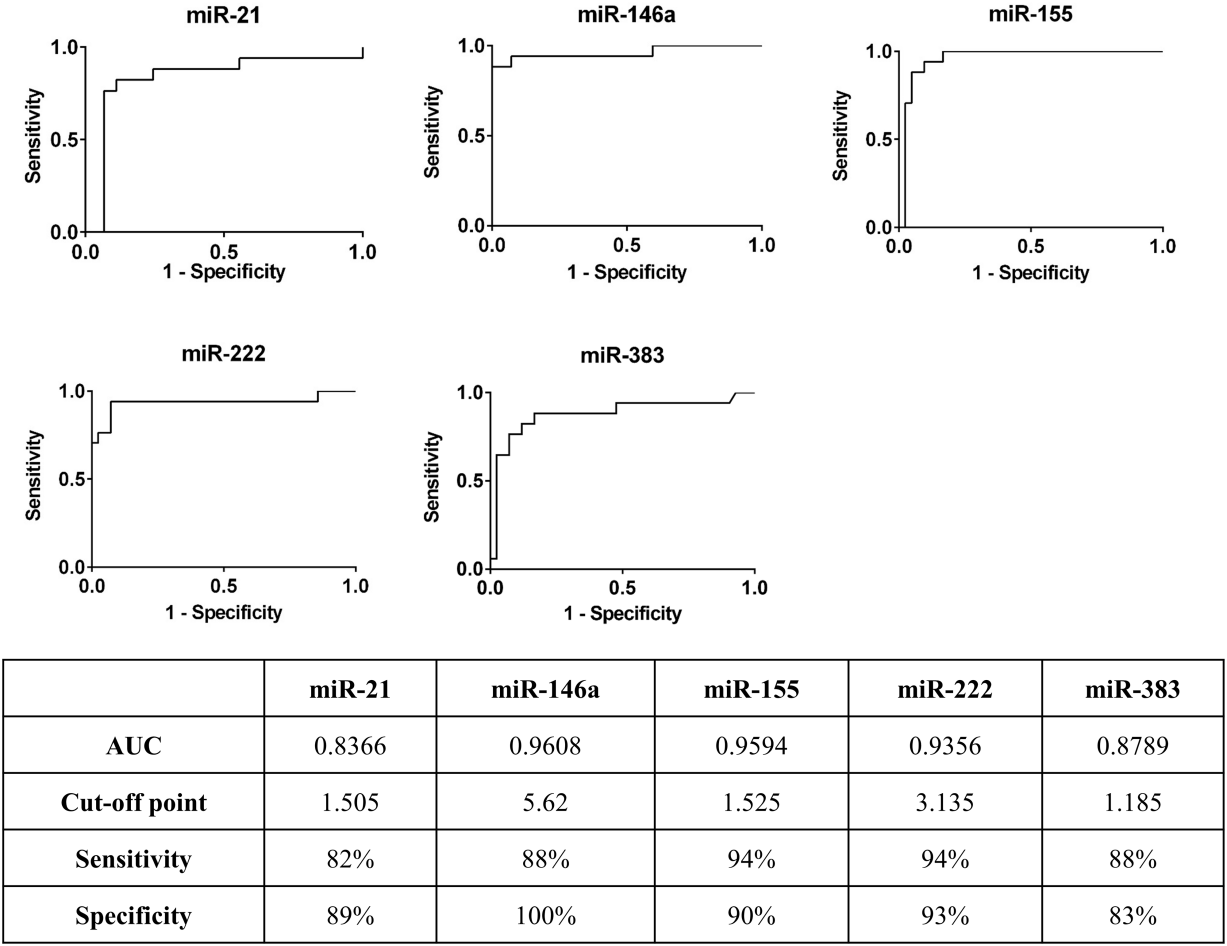
Mastitis diagnostic values of miRNA in milk quantified using qPCR. The Youden index was applied to determine the optimal cut-off point, sensitivity and specificity. AUC: area under the curve.

Receiver operating characteristic curve analysis of relative expression levels of five miRNA was performed to evaluate the ability of the miRNA to distinguish between the CMT+ and normal groups (Fig 3). Area under the curve analysis and the Youden index were applied to determine the optimal cut-off point, sensitivity and specificity of each miRNA [44]. We found that miR-146a, miR-155 and miR-222 had high predictive values (0.9 < AUC < 1); and miR-21 and miR-383 had moderate predictive values (0.7 < AUC < 0.9). miR-21, miR-146a, miR-155, miR-222, and miR-383 had sensitivity of 82%, 88%, 94%, 94%, and 88%, and specificity of 89%, 100%, 90%, 93%, and 83% in differentiating CMT+ milk from normal milk, respectively.

### Analysis of miRNA with altered expression level in CMT+ milk by digital PCR

**Fig 4:**
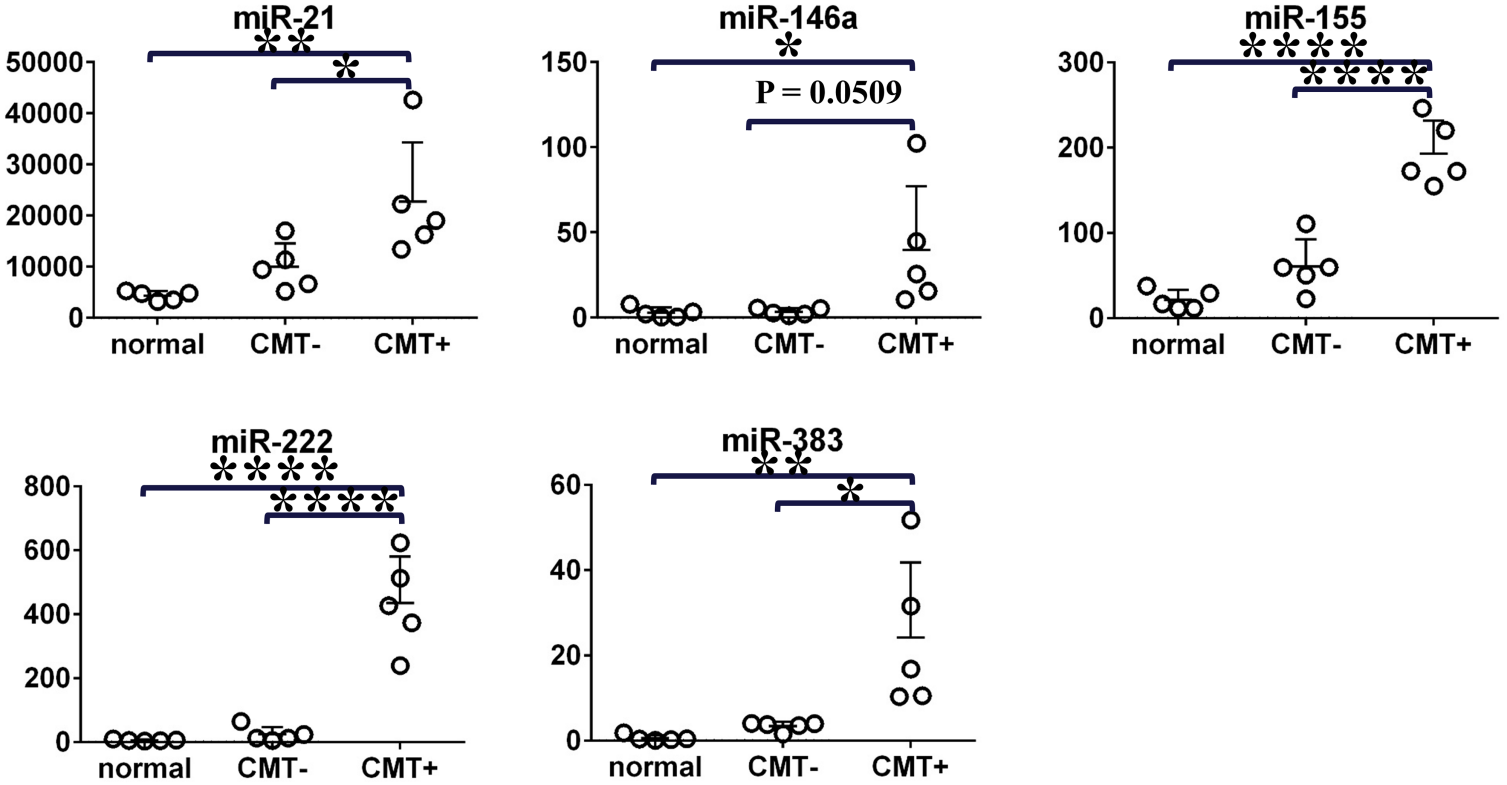
Digital PCR system quantification of expression levels of five miRNA in milk. Lower bars indicate mean values with vertical standard deviation bars. The y-axes represent copies/μL assessed by digital PCR (One-way ANOVA followed by Tukey's test, *P < 0.05, **P < 0.01, ****P <0.0001).

We selected the miRNA that were significantly upregulated in the CMT+ group (miR-21, miR-146a, miR-155, miR-222, and miR-383) for QuantStudio 3D Digital PCR System analysis. We analyzed milk from five normal cows and five mastitis-affected cows (including five CMT− quarters and five CMT+ quarters) in this validation study. The expression levels of the five miRNA were significantly higher in the CMT+ group than in the normal group. The miRNA were also significantly upregulated in the CMT+ group compared with the CMT− group, except for miR-146a (P = 0.0509). The expression levels of all miRNA did not differ between the CMT− and normal groups (Fig 4).

**Fig 5:**
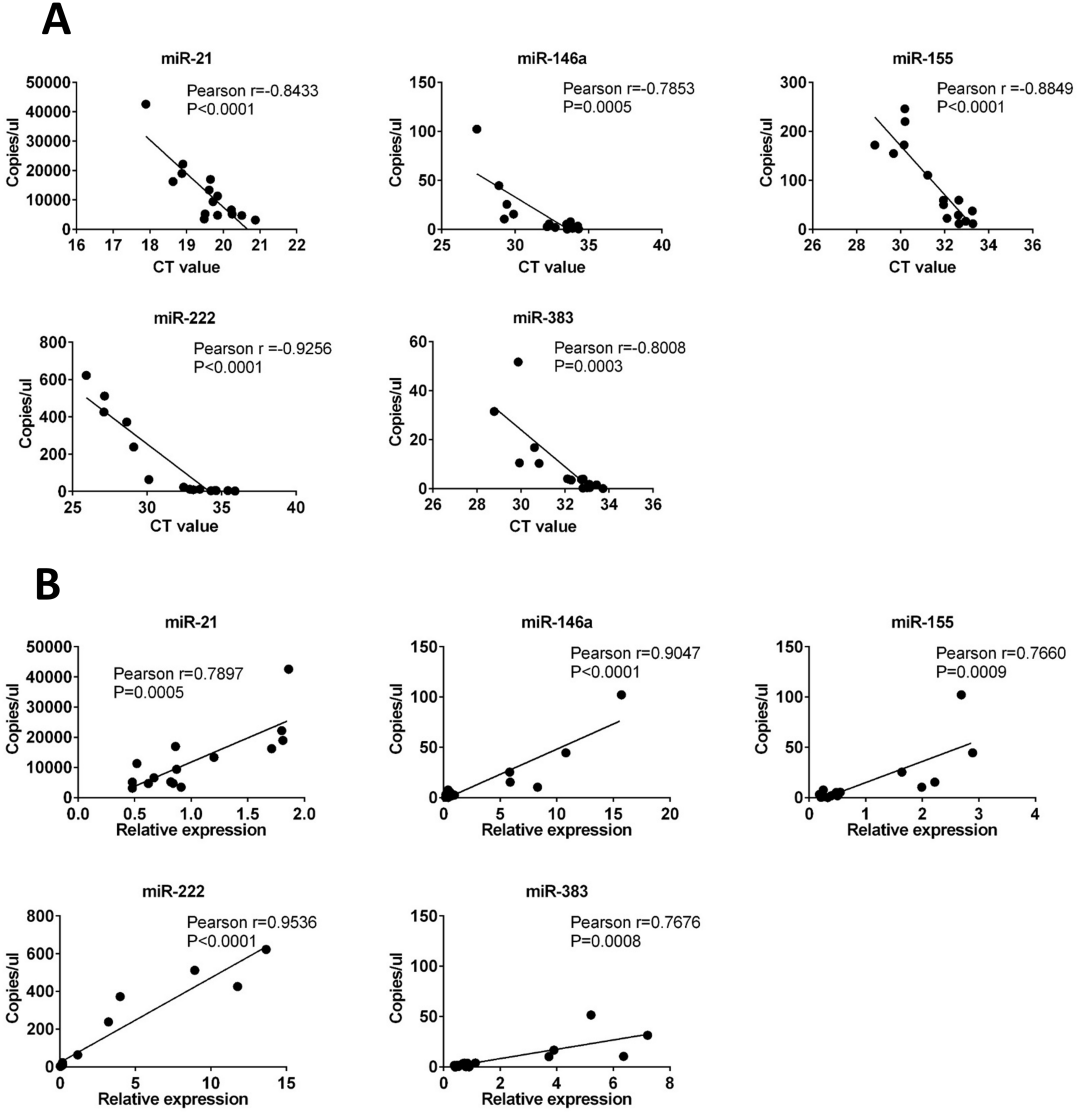
Relationship between the results of qPCR and digital PCR. **(A)** copies/μL obtained via digital PCR and CT value obtained via qPCR. **(B)** copies/μL obtained via digital PCR and relative expression level obtained via qPCR.

Pearson’s correlation analysis was applied to assess the relationship between the qPCR and digital PCR results. We found a strong negative correlation between the Ct values obtained via qPCR and the values for copies/μL obtained via digital PCR (the Pearson r values for miR-21, miR-146a, miR-155, miR-222, and miR-383 were −0.8433, −0.7853, −0.8849, −0.9256, and −0.8008, respectively)(Fig 5A), and a strong positive correlation between relative expression levels obtained via qPCR and the values for copies/μL obtained via digital PCR (the Pearson r values for miR-21, miR-146a, miR-155, miR-222, and miR-383 were 0.7897, 0.9047, 0.7660, 0.9536, and 0.7676, respectively)(Fig 5B). These results suggest that chip-based QuantStudio 3D Digital PCR System could be a tool for quantification of miRNA in milk.

### Discussion

Dysregulation of miRNA expression level has been shown to play a role in inflammatory diseases [47]. Previous studies have demonstrated that miRNA expression level in mastitis-affected cows is altered in mammary epithelial cells [48–50], monocytes [51], milk exosome [52], and mammary gland tissue [41, 53]. Our study showed that expression level of miRNA was altered in CMT+ milk, suggesting that miRNA may play a role in bovine mastitis. Our findings suggest the potential for development of molecular biology-based biomarkers for bovine mastitis-affected milk. We further verified the miRNA upregulation using a QuantStudio 3D Digital PCR System, and obtained reproducible results. To the best of our knowledge, this is the first report to compare miRNA in milk from mastitis-affected and normal cows.

Liquid biopsy allows diagnosis of diseases in noninvasive, safe, and fast way using biomarkers isolated from body fluids, such as blood and urine [9]. Circulating miRNA have been proposed to have either diagnostic or prognostic value in various types of human cancer [54]. We evaluated the suitability of miRNA in milk as liquid biopsy biomarkers using receiver operating characteristic analysis. Our results showed that several miRNA had high predictive values (AUC greater than 0.83) and sensitivity and specificity greater than 80% in differentiating CMT+ milk from normal cow milk. These results demonstrate the potential of miRNA in milk for use as a liquid biopsy biomarker for mastitis.

Previous studies have demonstrated that droplet digital PCR can measure expression level of miRNA in body fluids, and that these miRNA can serve as diagnostic biomarkers. For example, miRNA in serum can be used as a biomarker in breast cancer diagnosis [55], and miRNA in sputum and plasma can be used for lung cancer diagnosis [56, 57]. We used a QuantStudio 3D Digital PCR System to measure miRNA expression levels in milk, and compared the results with those of qPCR. Direct comparison of miRNA expression levels and CT values obtained via qPCR with copy numbers obtained via digital PCR for the same sample set demonstrated a strong correlation between the two methods. A previous study also indicated high correlation between copy numbers obtained via digital PCR and expression levels determined by qPCR across serially diluted samples [57]. In this study, we demonstrated that the results of qPCR were reproducible using QuantStudio 3D Digital PCR. Therefore, the chip-based QuantStudio 3D Digital PCR System could be a tool for quantification of miRNA in milk for diagnosis of bovine mastitis.

There are some of limitations to our study. First, further evaluation in large cohorts of the miRNA identified in this study is required before they could be used as robust biomarkers. Second, we used the California mastitis test (CMT), which is based on the somatic cell count of milk to detect mastitis. In addition to inflammation, there are many other factors that could influence milk somatic cell count, such as seasonal effects and physiological or environmental stress [18, 19].

In conclusion, miR-21, miR-146a, miR-155, miR-222, and miR-383 expression levels were significantly upregulated in the CMT+ milk. Our findings suggest that inflammation-related miRNA expression level in the bovine milk was affected by mastitis, and miRNA in milk have potential for use as biomarkers of bovine mastitis.

## Supporting information

S1 Table The detailed sample information of normal and mastitis cows. (XLSX)

S2 Table The detailed sample information of normal, CMT− and CMT+ quarters. (XLSX)

S3 Table (A) CT values of miR-26b, miR-29b, miR-92a, miR-122, miR-125b, miR-222, miR-204, miR-205 and miR-383 in normal and mastitis cows. (B) CT values of miR-21-5p and miR-92a in normal and mastitis cows.
(XLSX)

S4 Table CT values of miRNA in normal, CMT− and CMT+ quarters. (XLSX)

S1 Fig Small RNA analysis results. The numbers indicate sample number.
(PDF)
