# Supplementary figures and images for: Inflammation-related microRNA expression level in the bovine milk is affected by mastitis
Source: PLoS One. 2017 May 17;12(5):e0177182. doi: 10.1371/journal.pone.0177182 (PMC5435311; doi:10.1371/journal.pone.0177182)

## S1 Fig. Small RNA analysis results.

The numbers indicate sample number.

### Negative Control

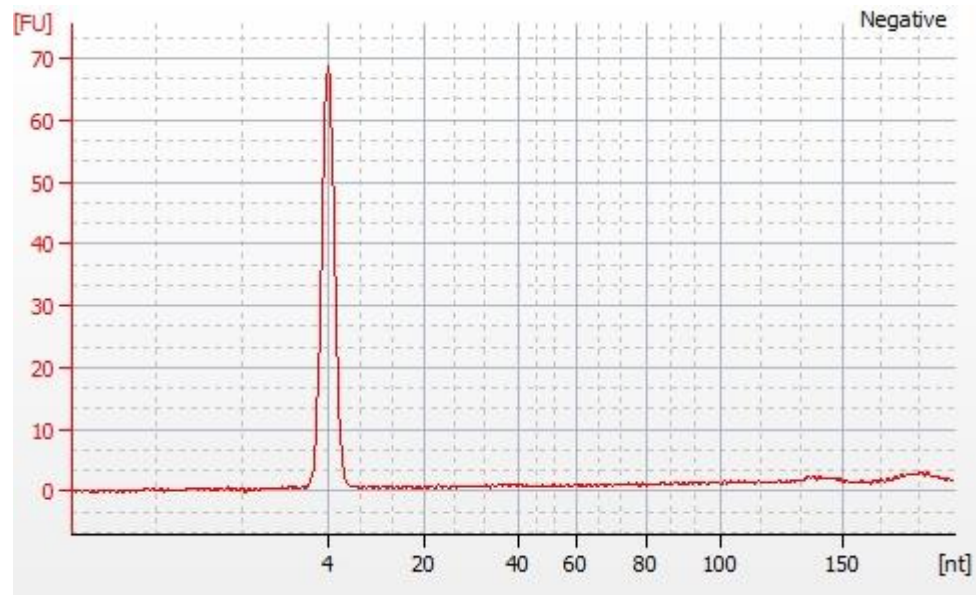

1

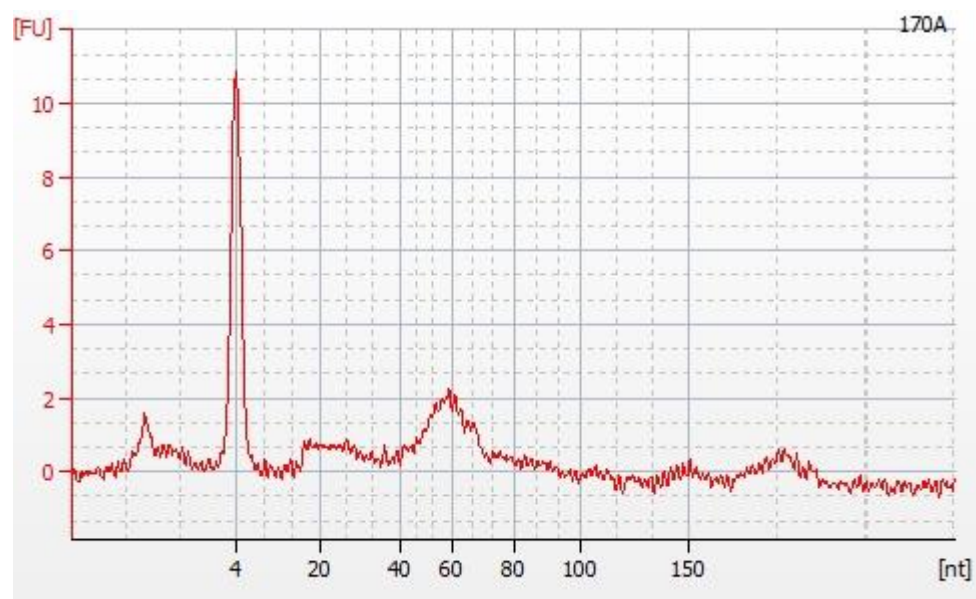

2

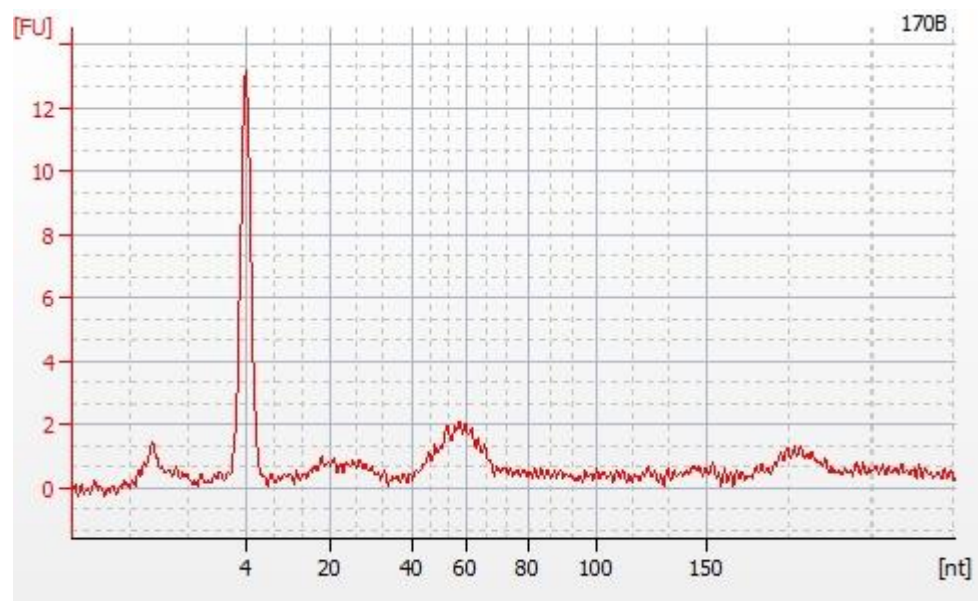

3

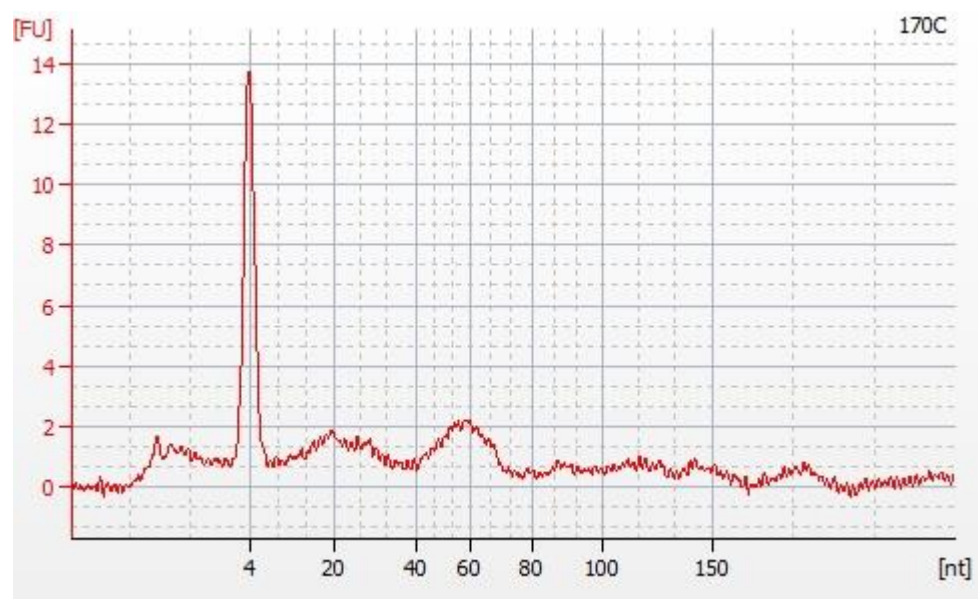

4

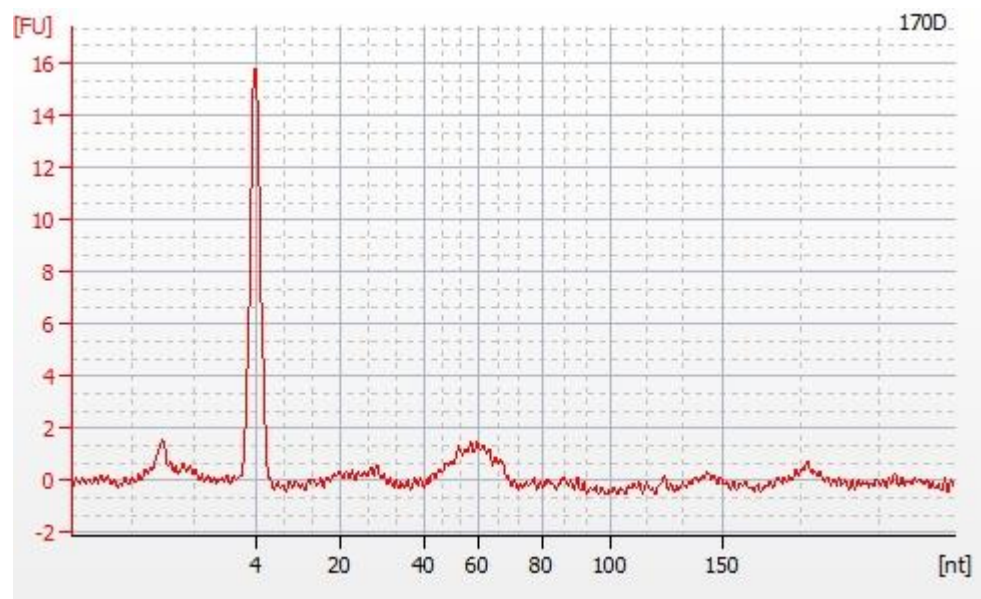

5

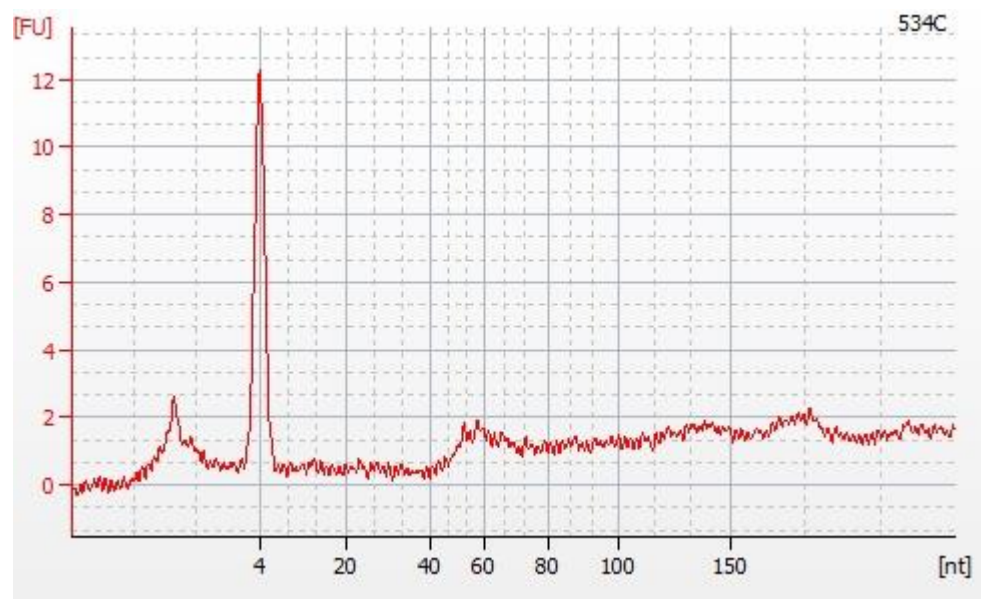

6

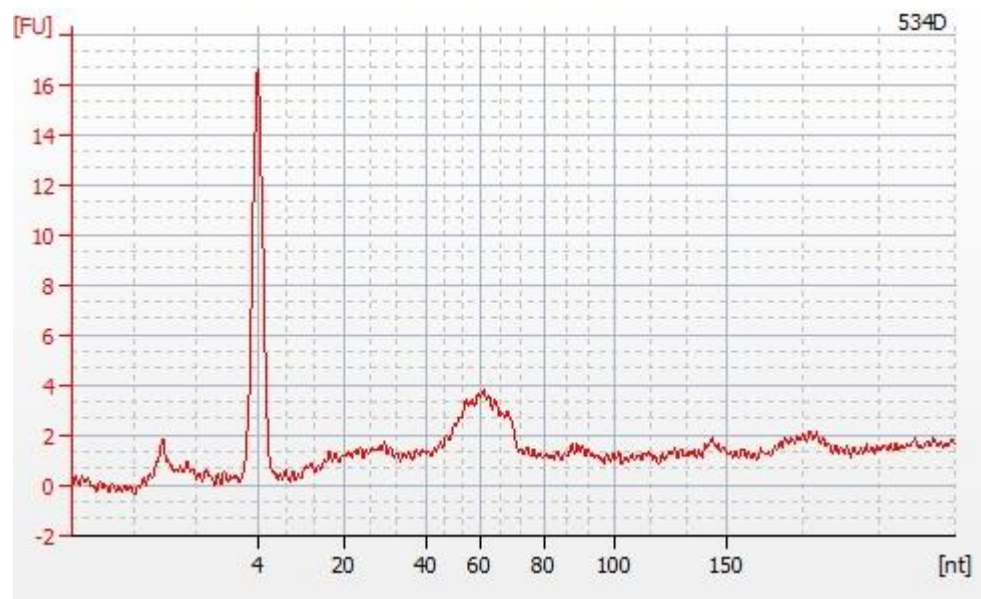

7

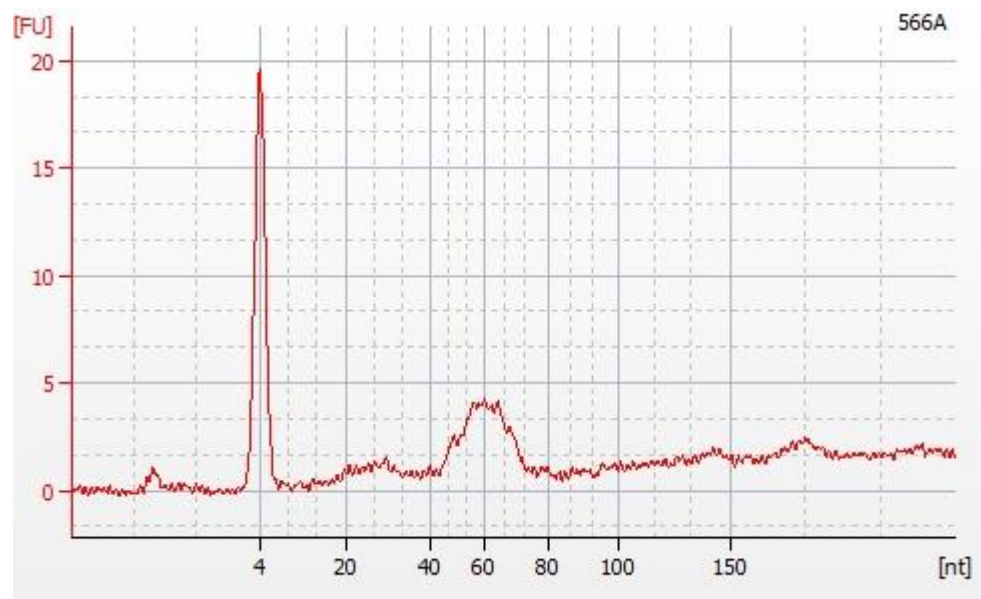

8

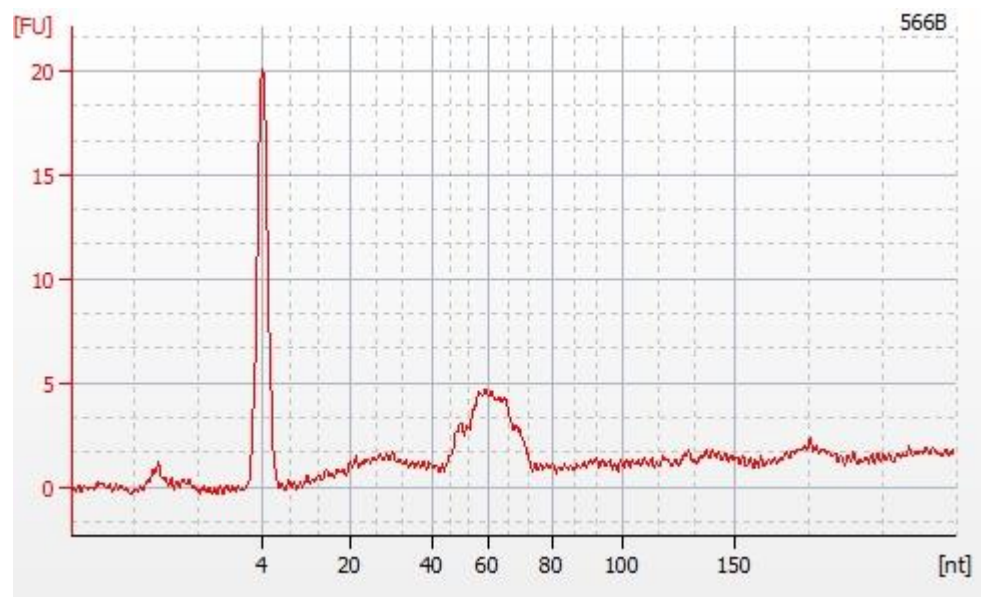

9

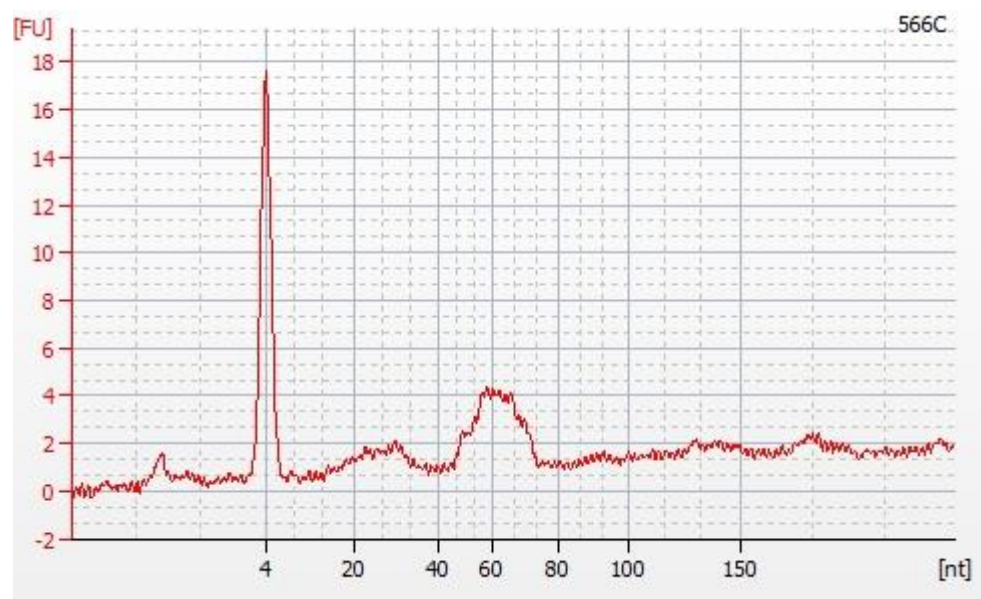

10

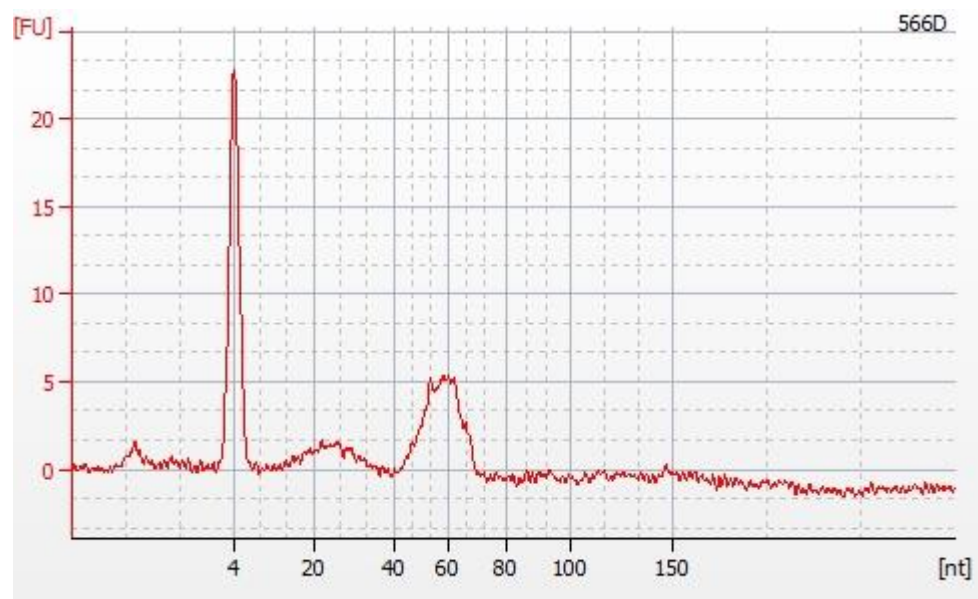

11

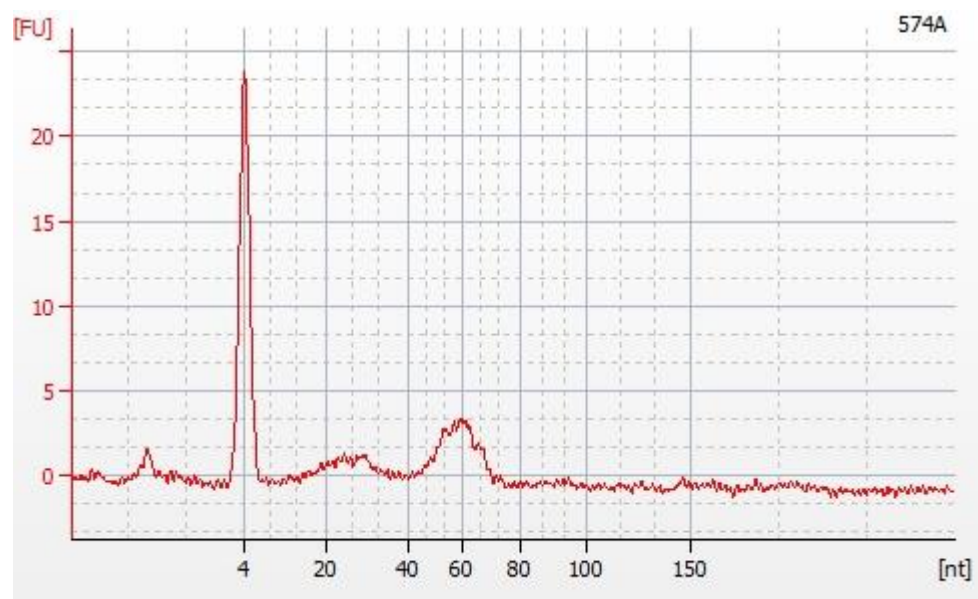

12

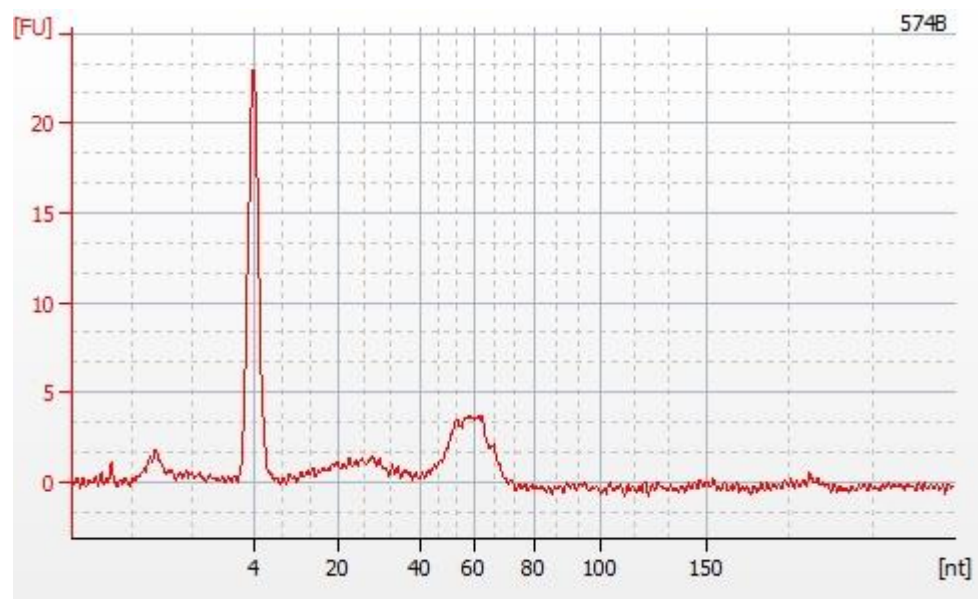

13

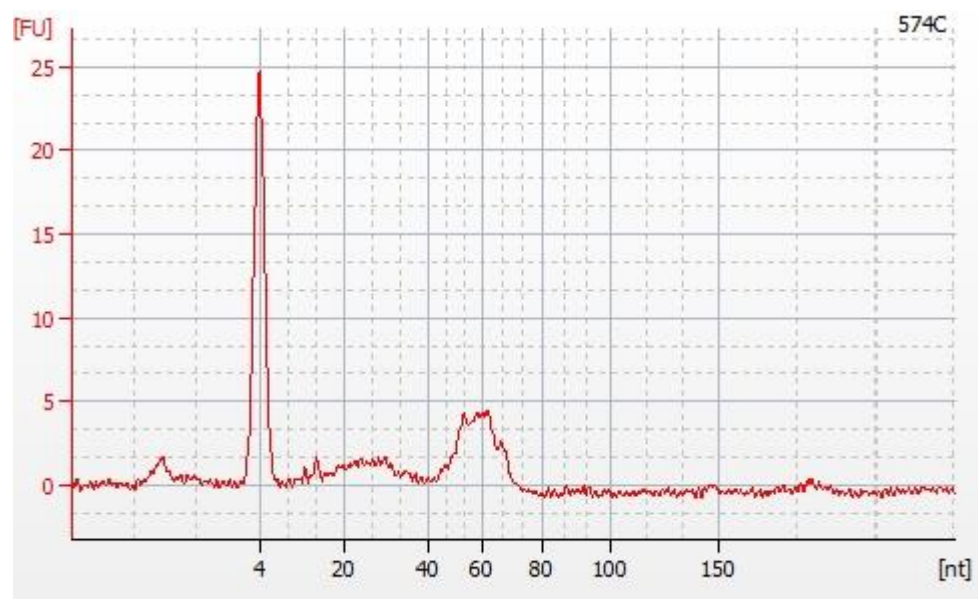

14

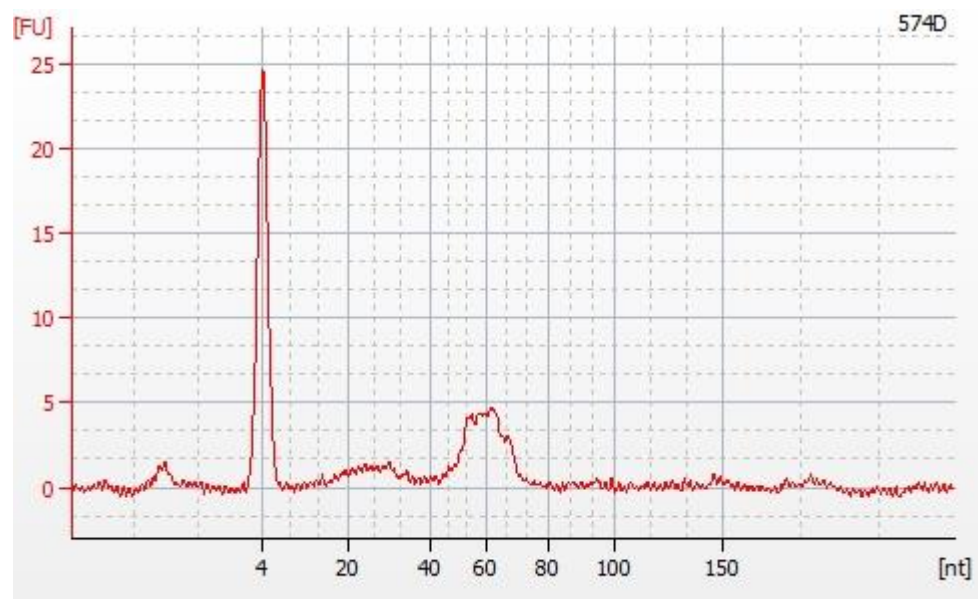

15

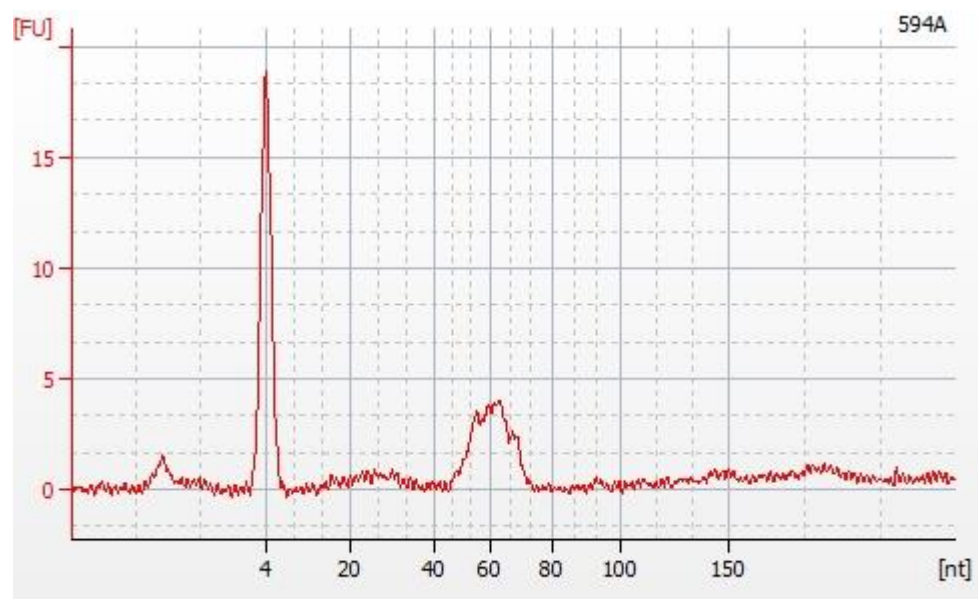

16

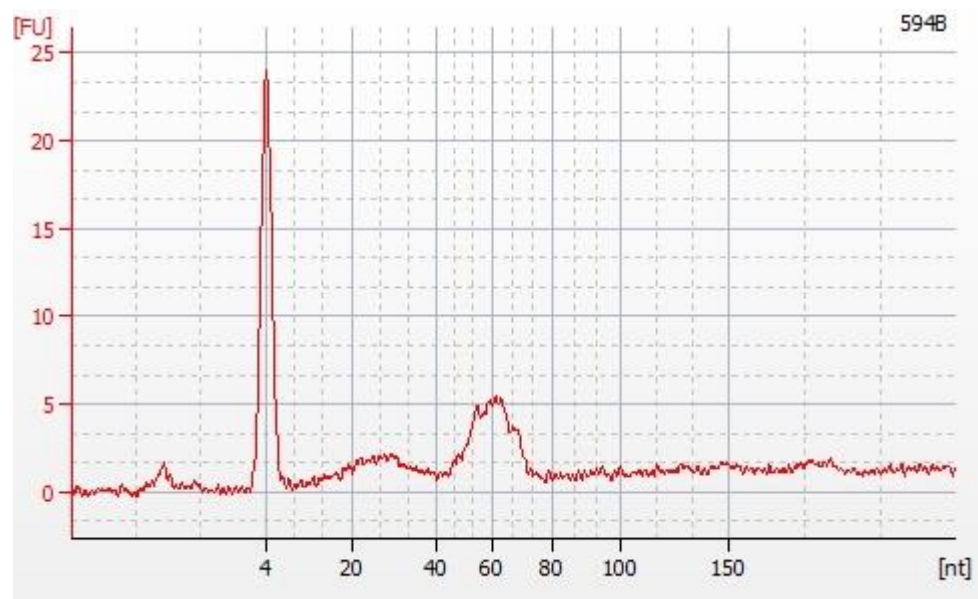

17

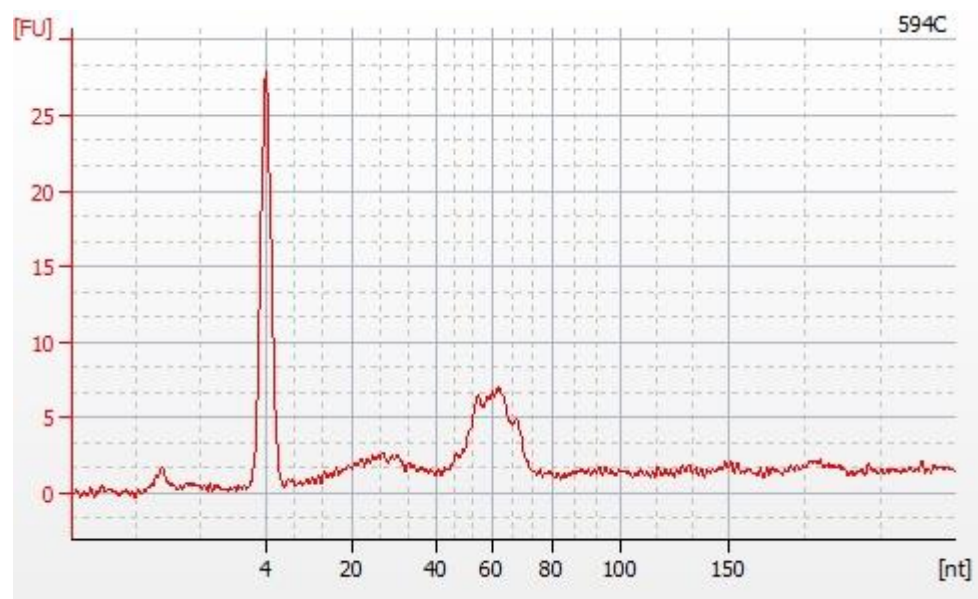

18

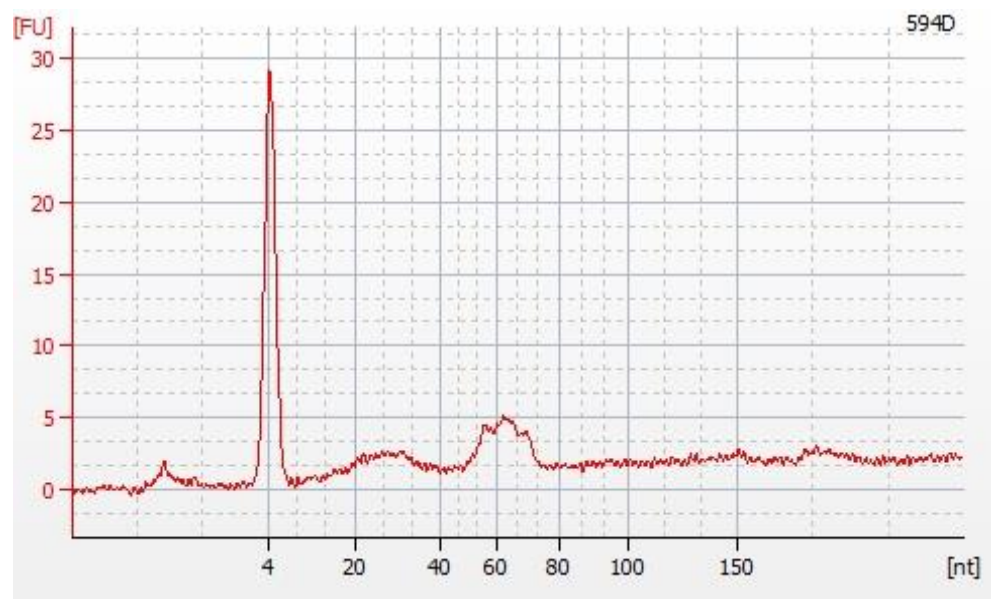

19

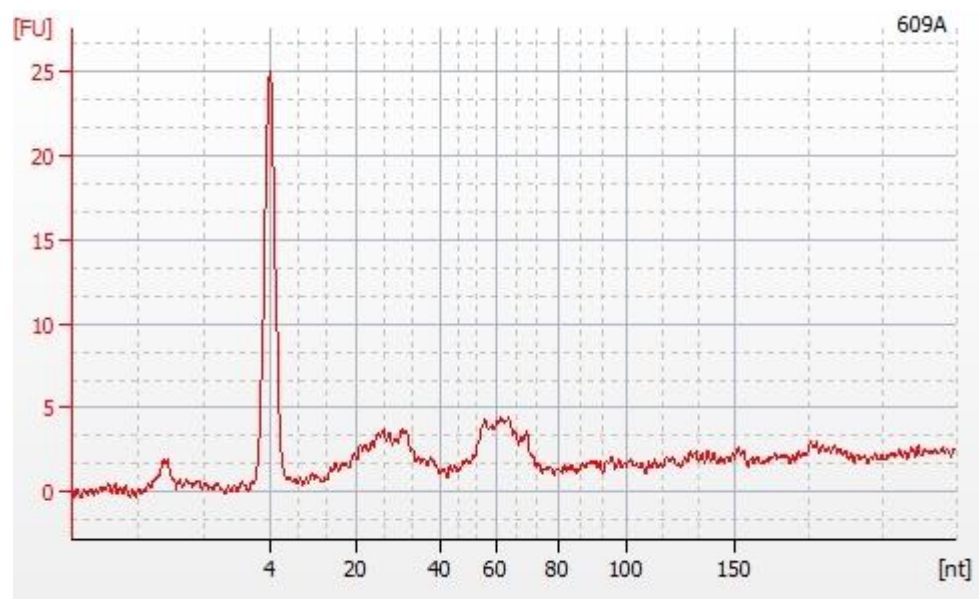

20

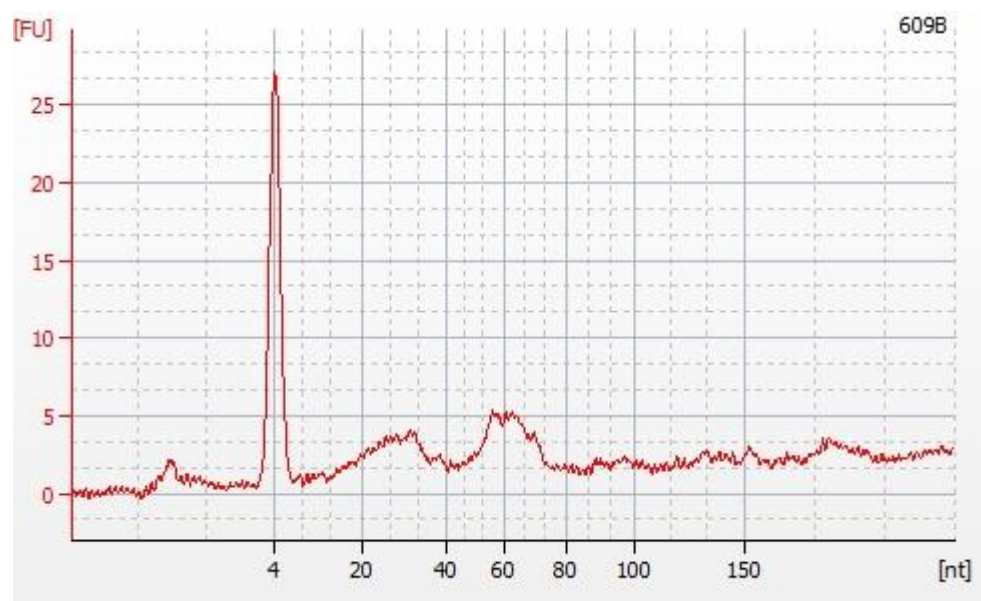

21

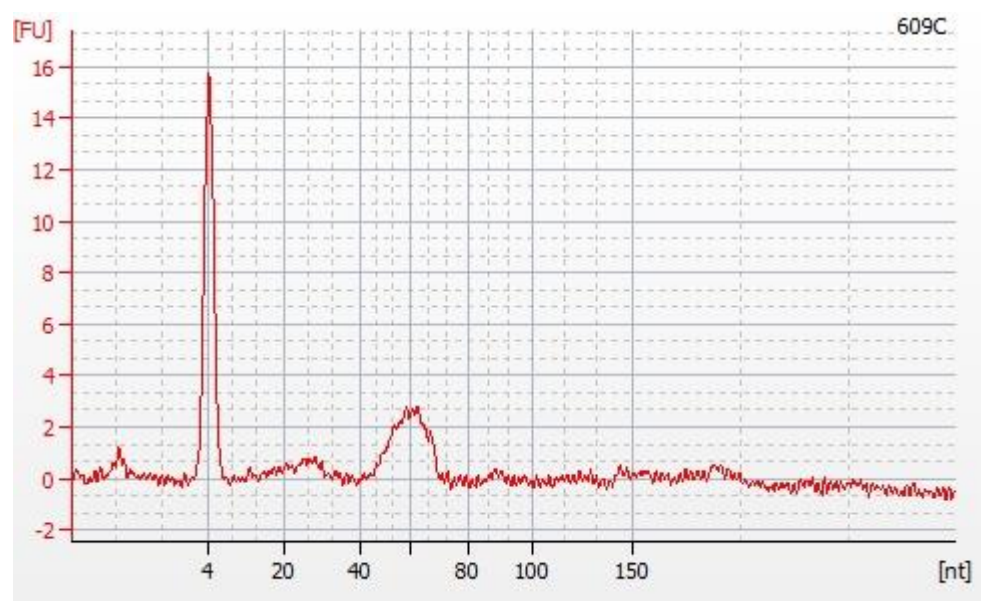

22

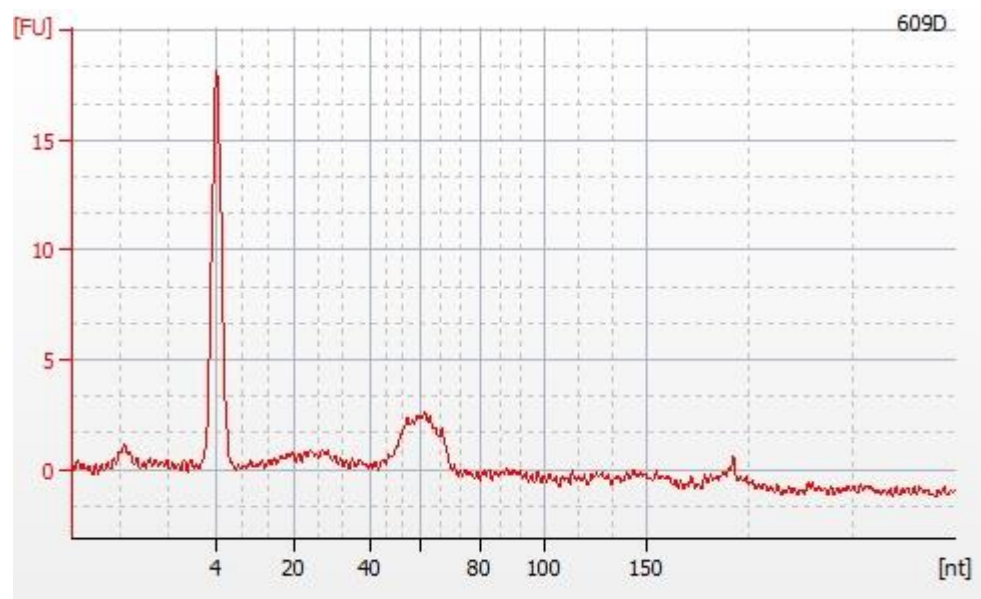

23

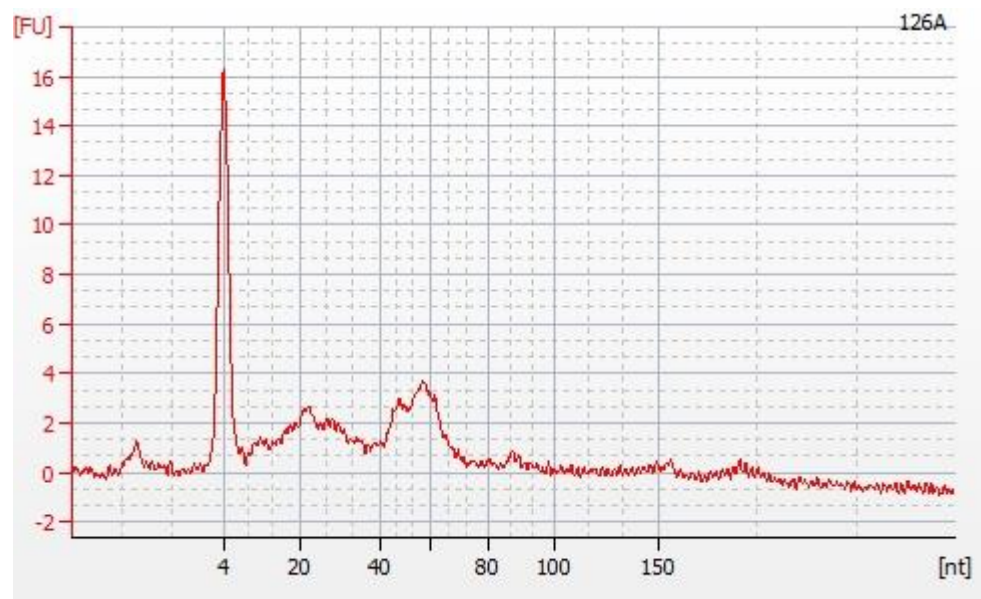

24

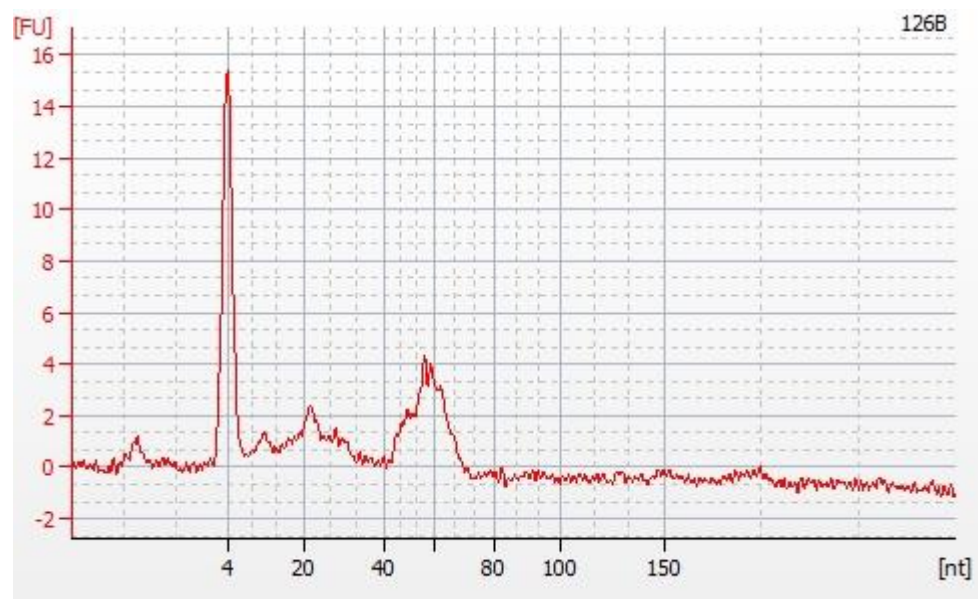

25

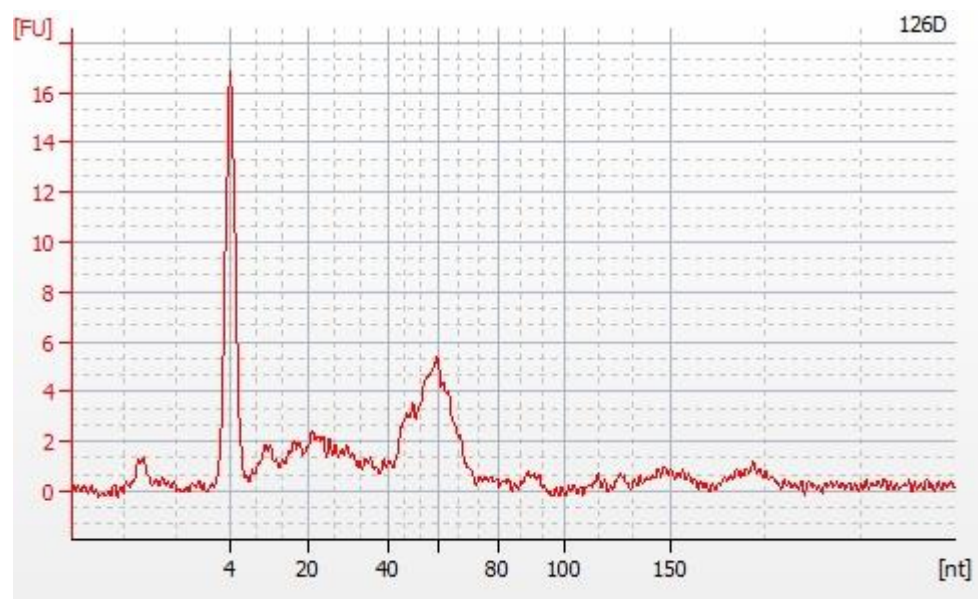

26

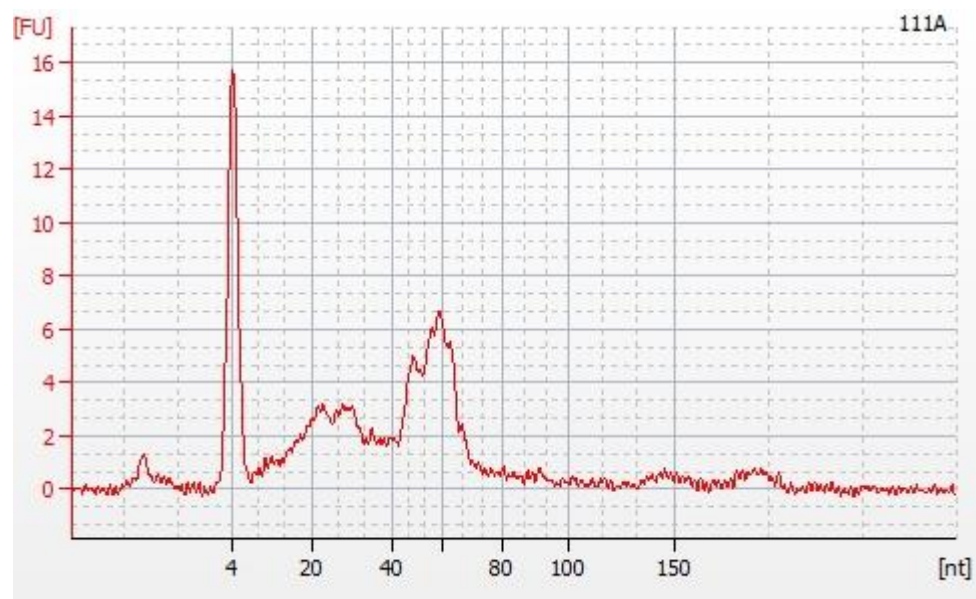

27

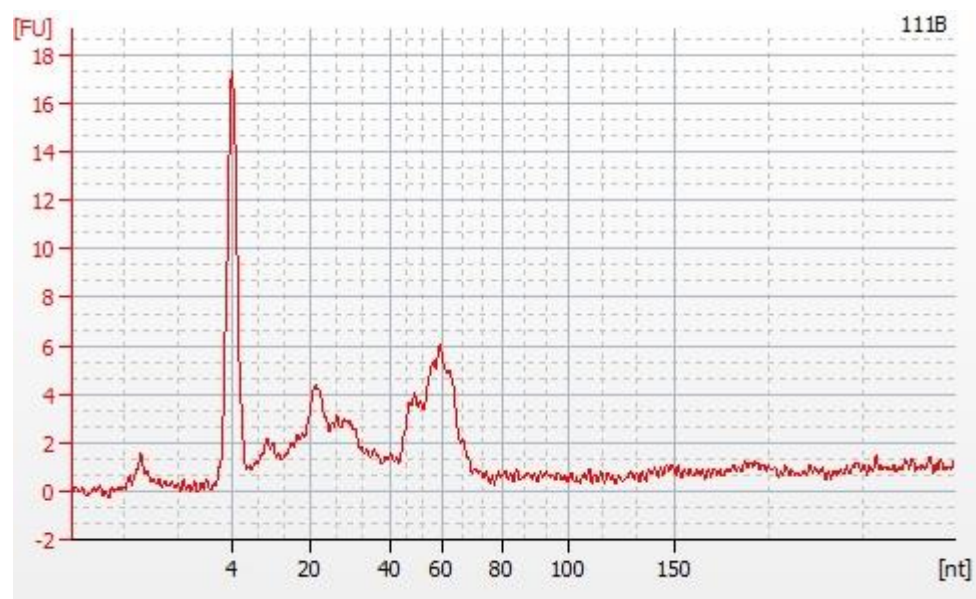

28

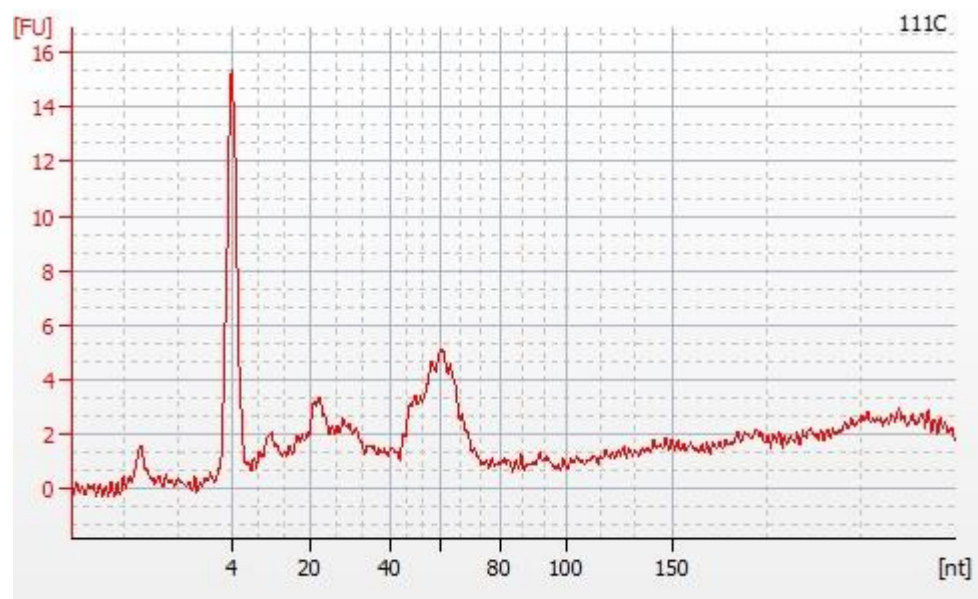

29

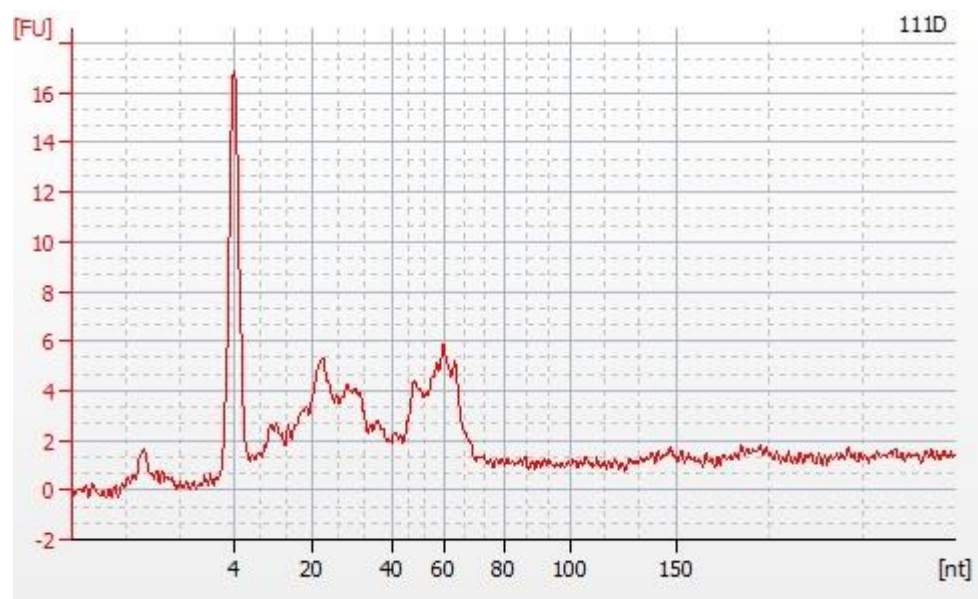

32

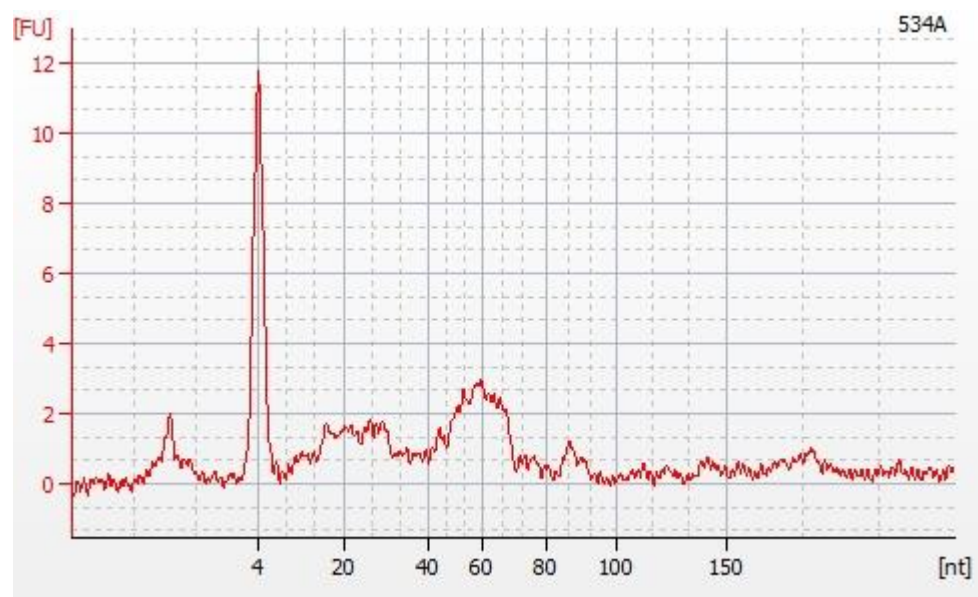

33

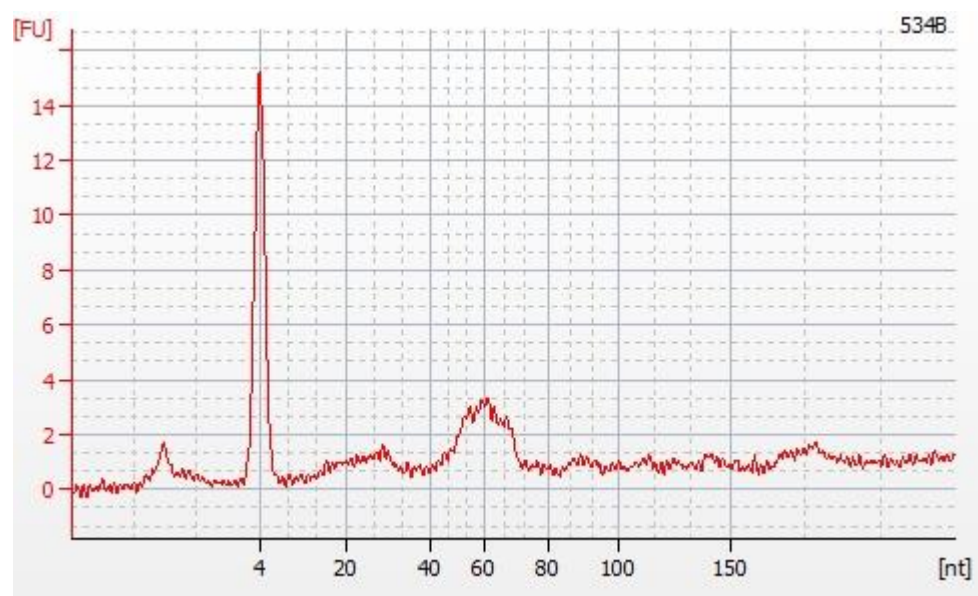

34

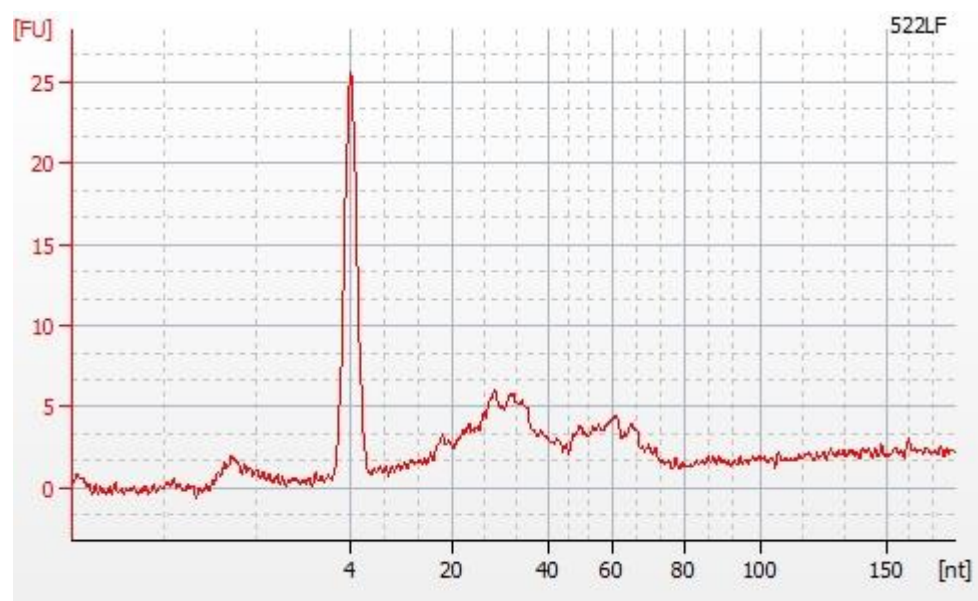

35

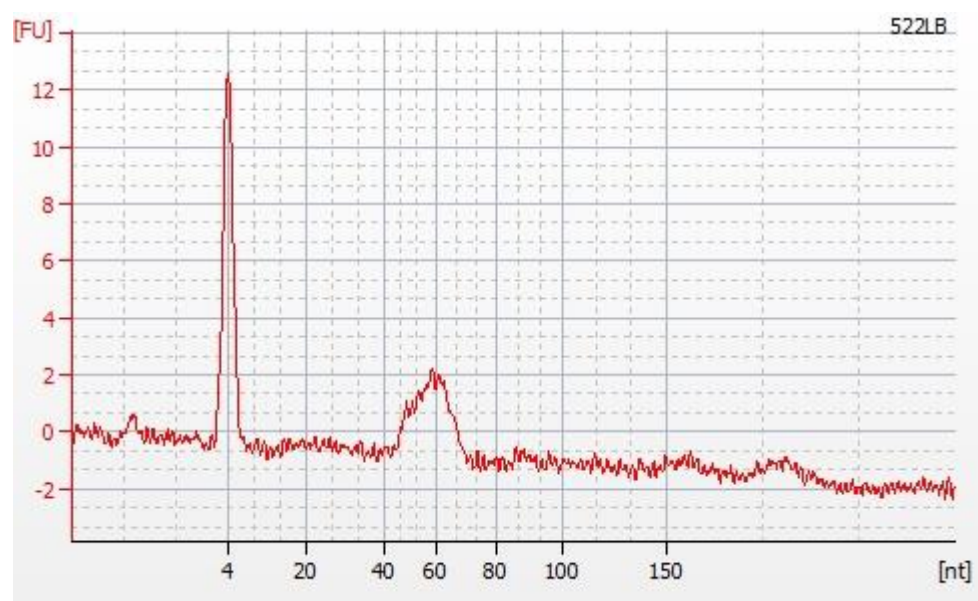

36

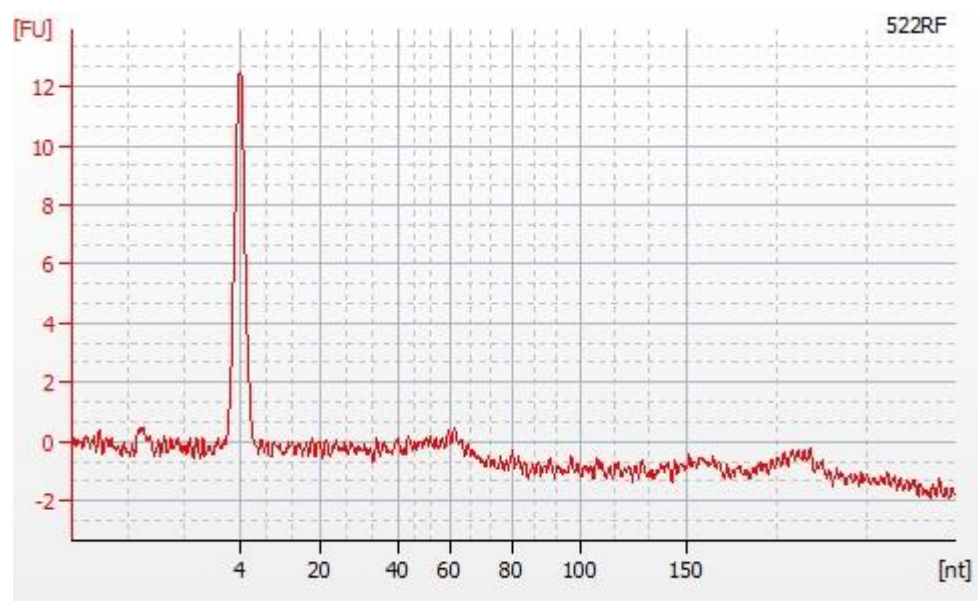

37

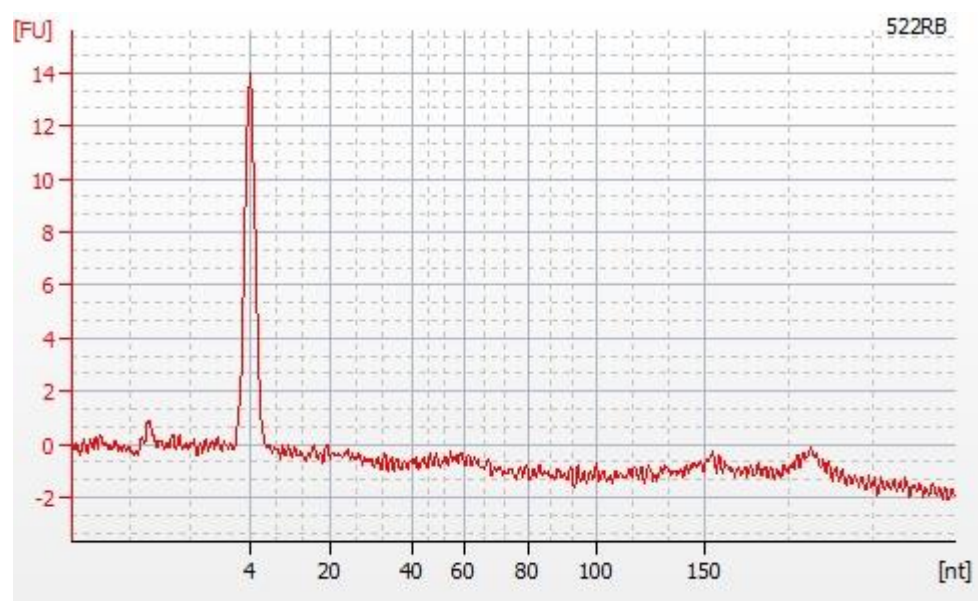

38

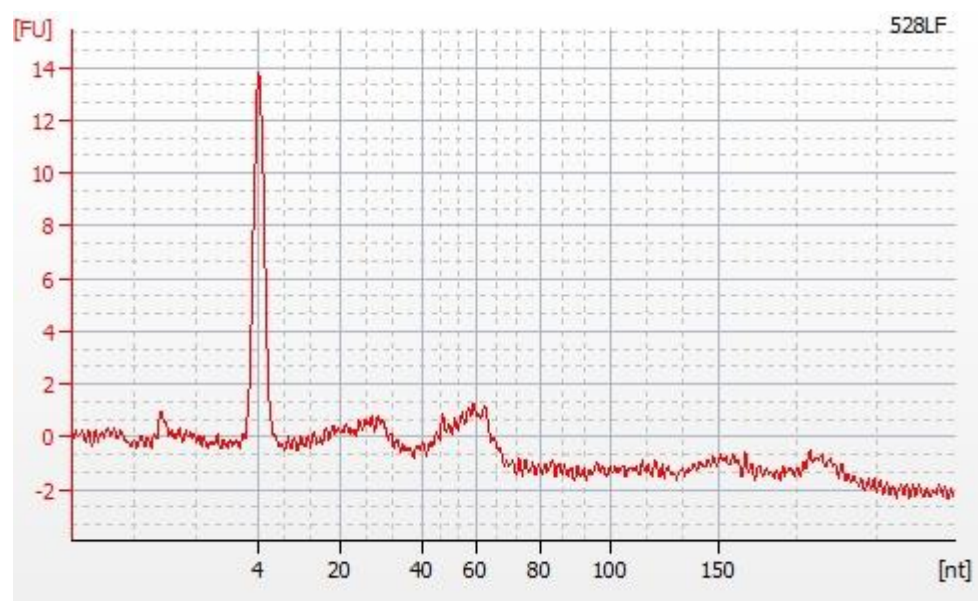

39

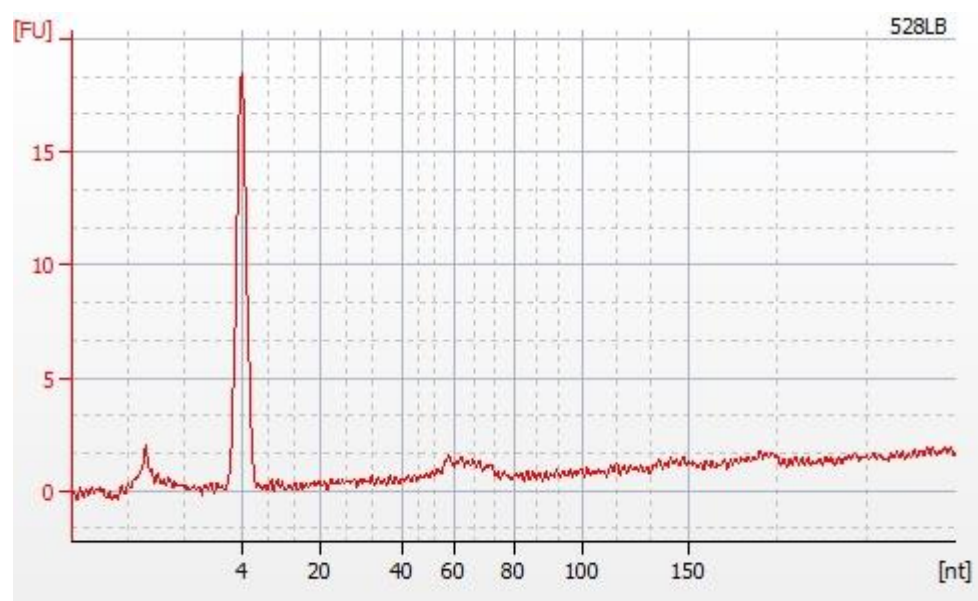

40

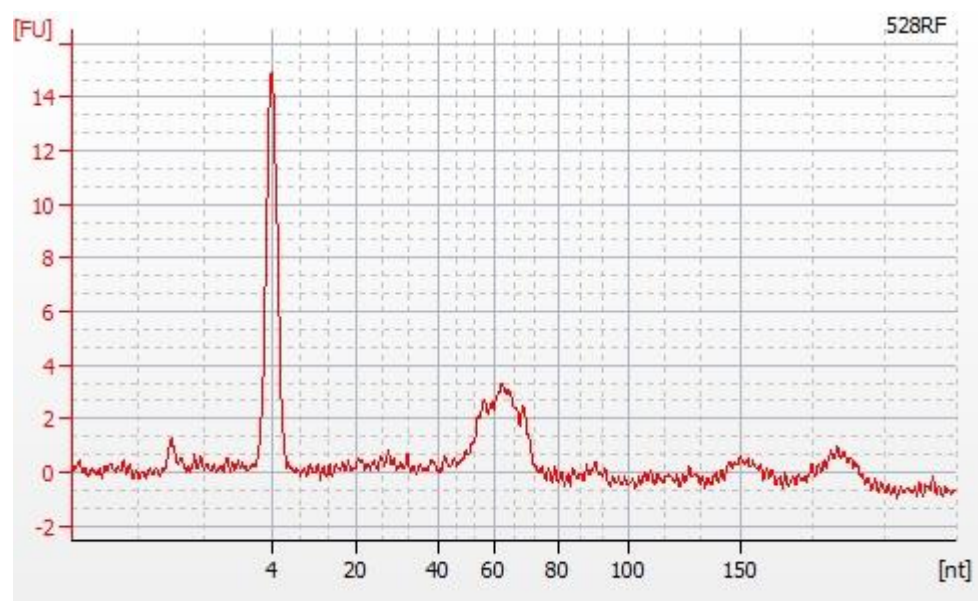

41

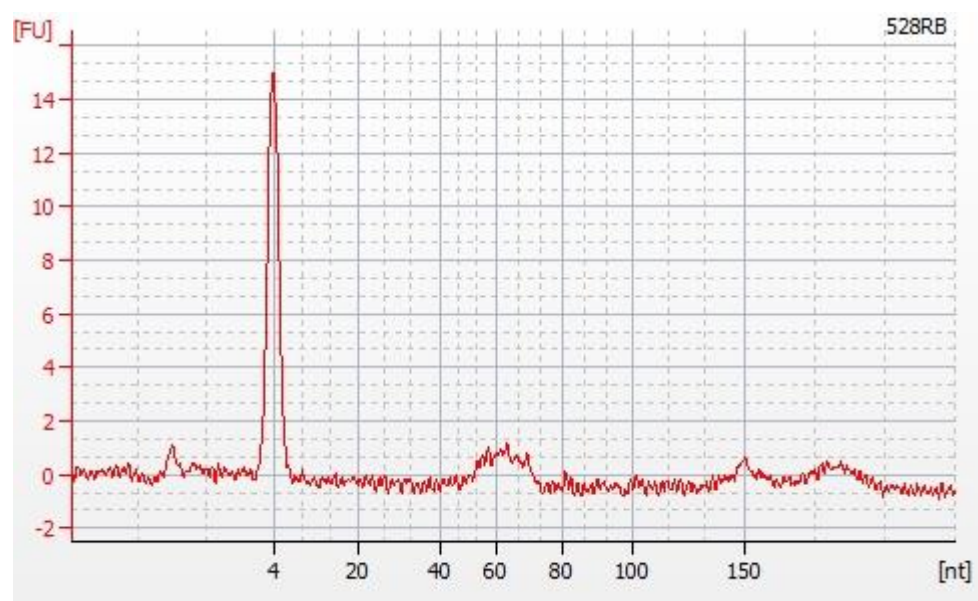

42

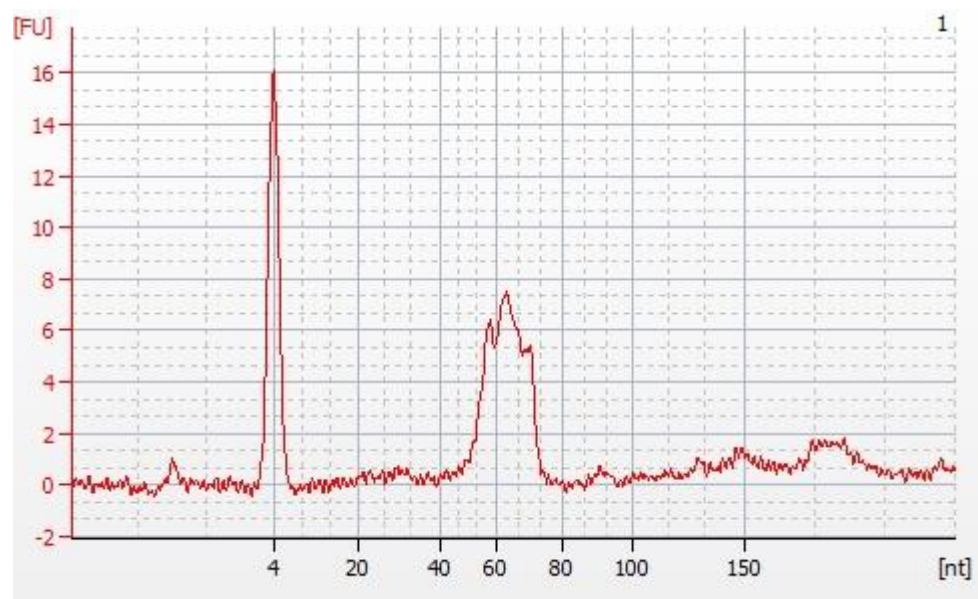

43

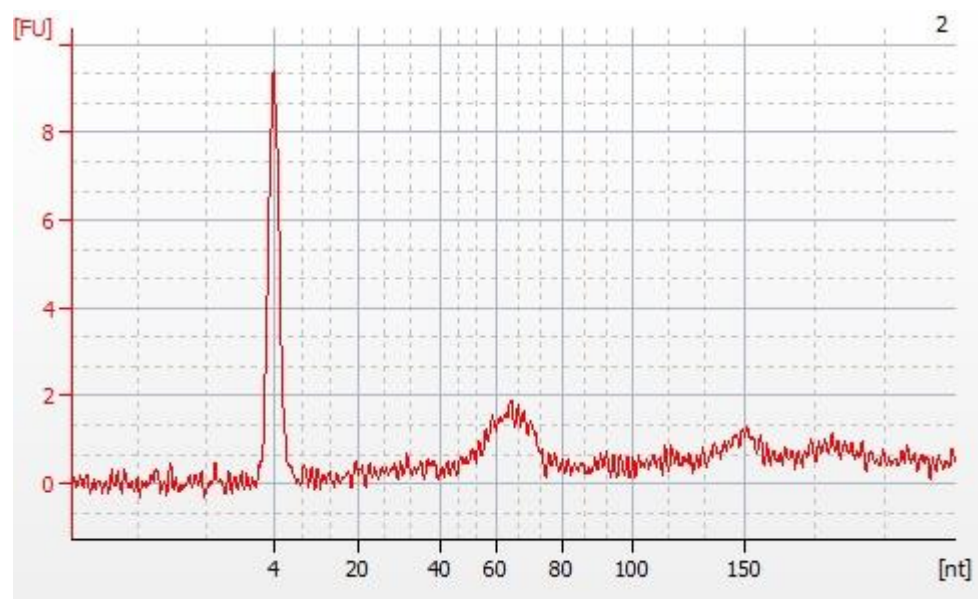

44

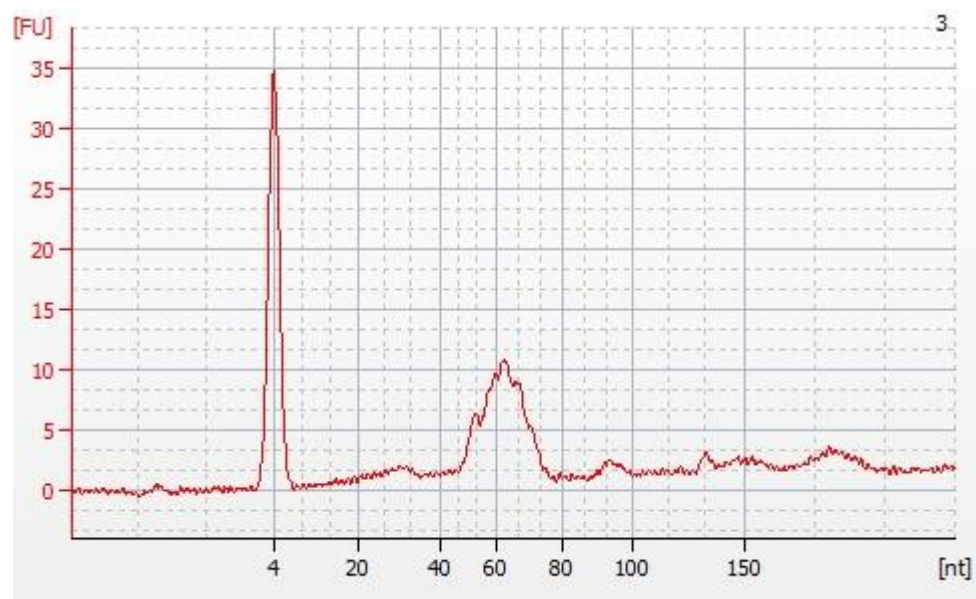

45

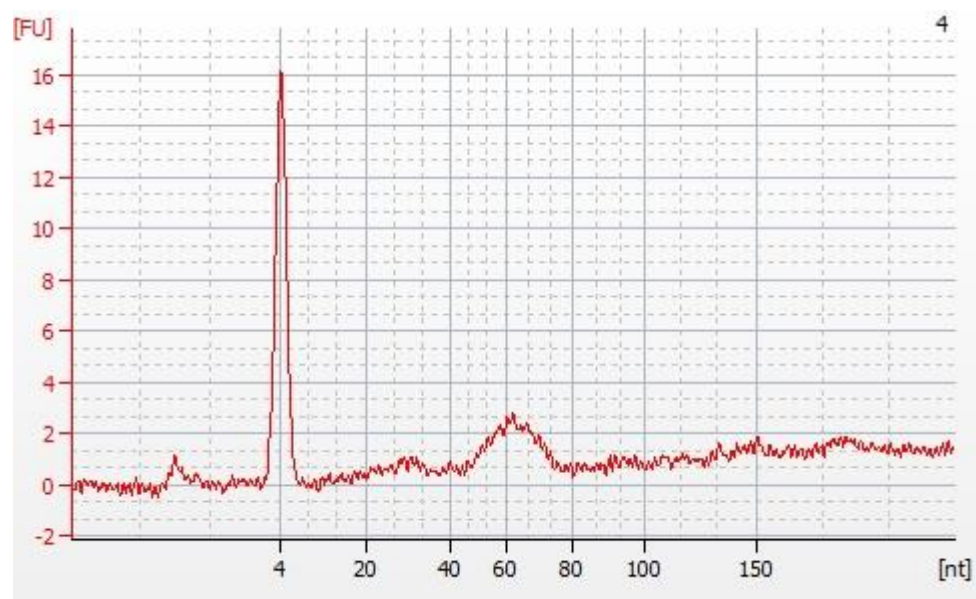

47

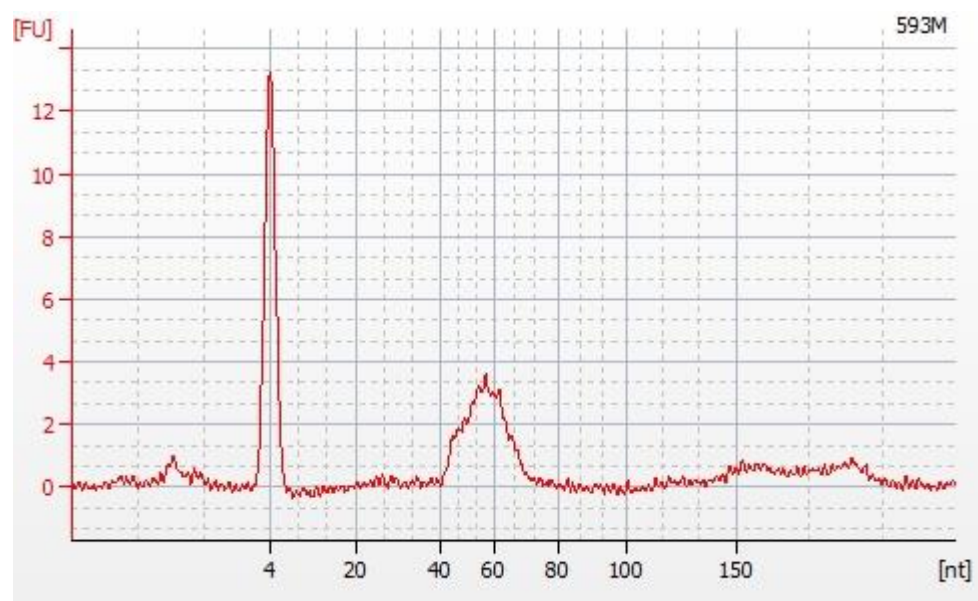

48

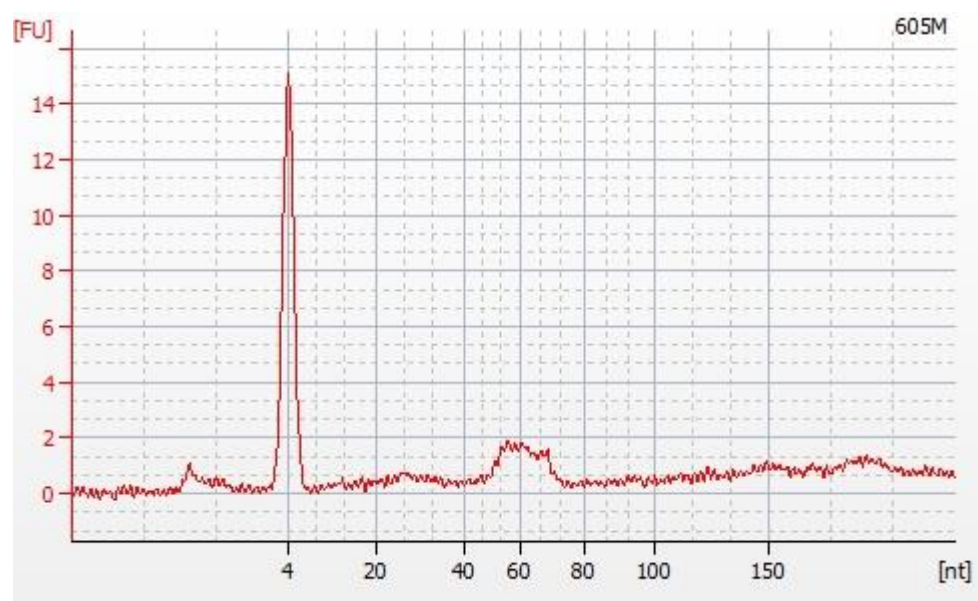

49

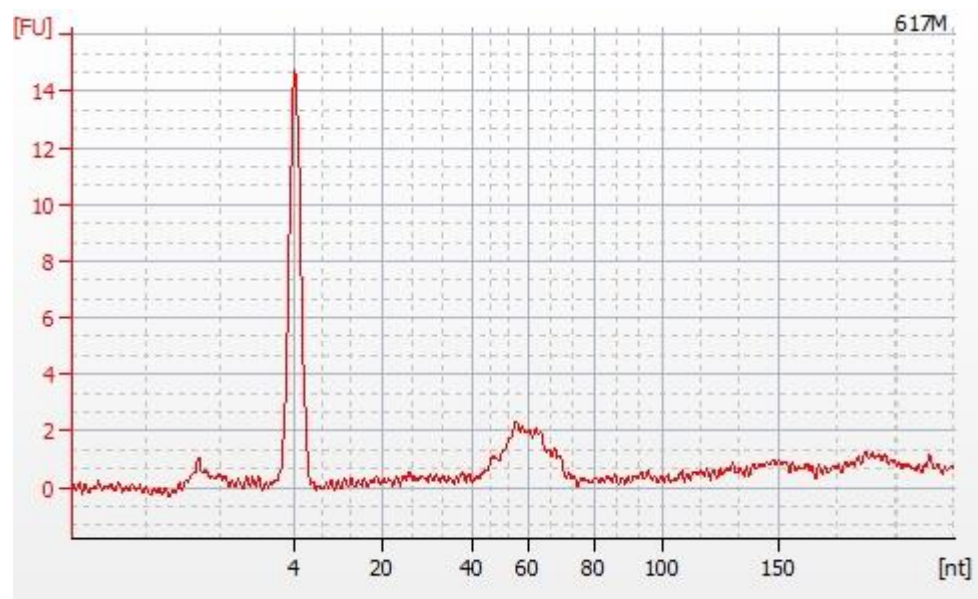

50

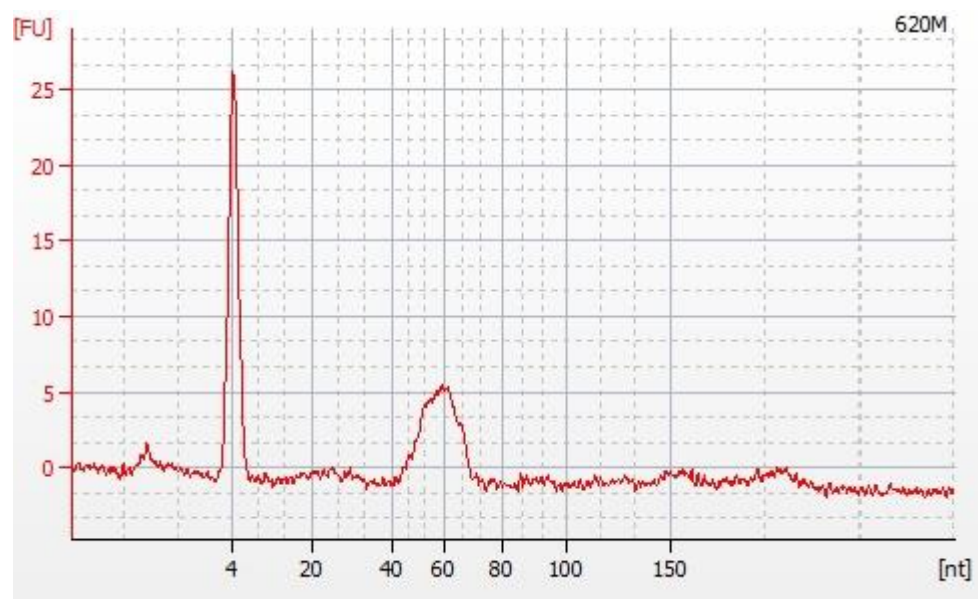

51

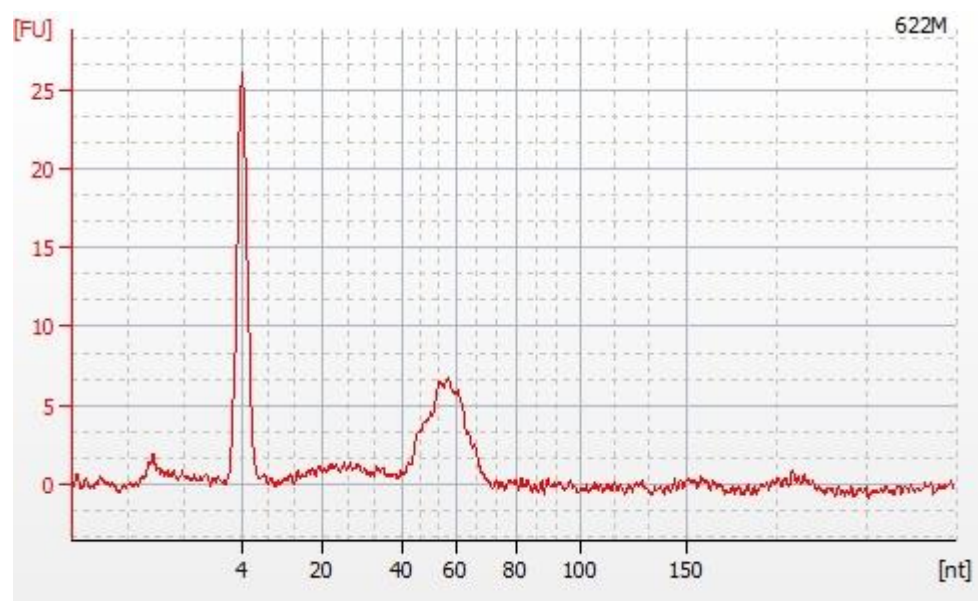

52

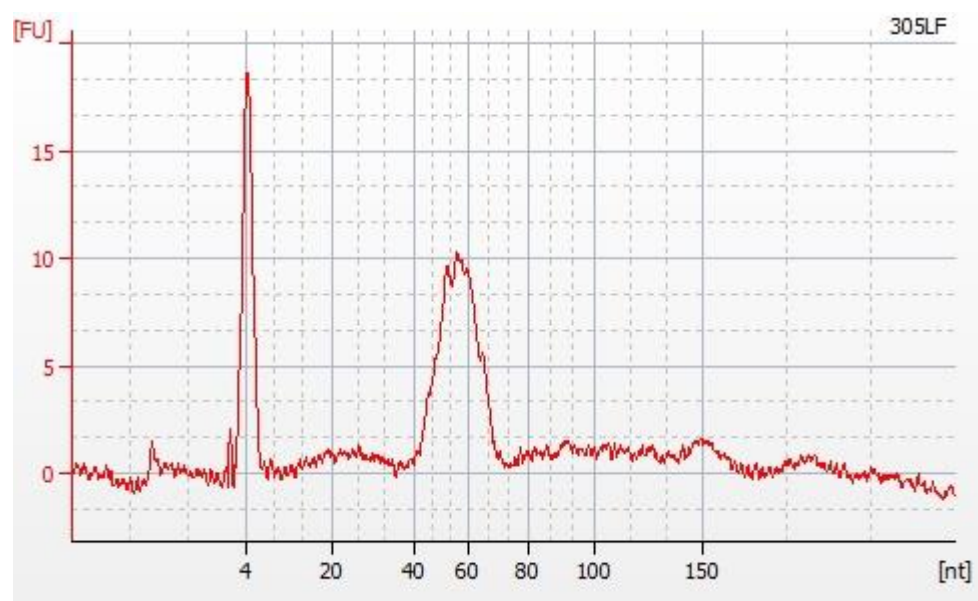

53

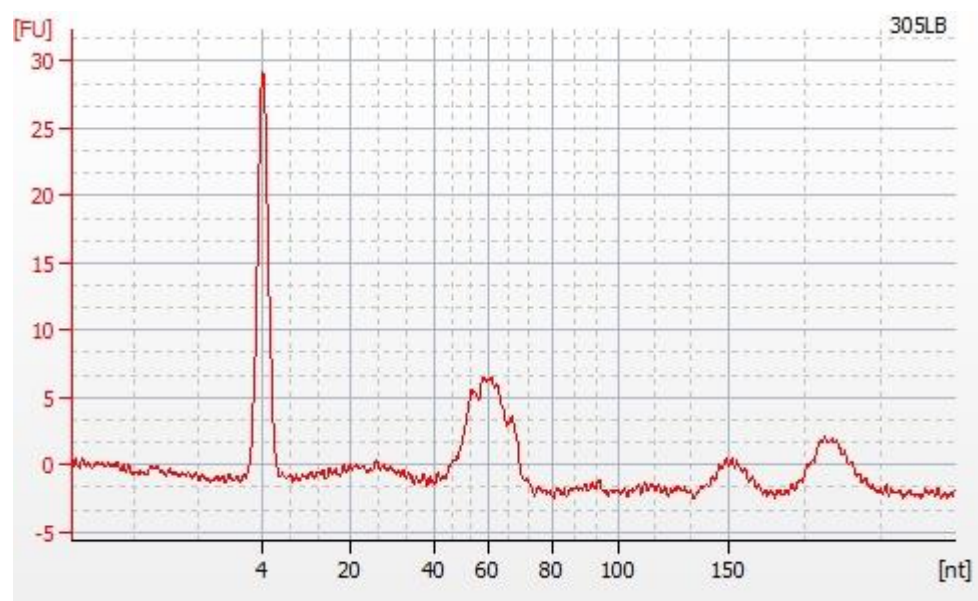

54

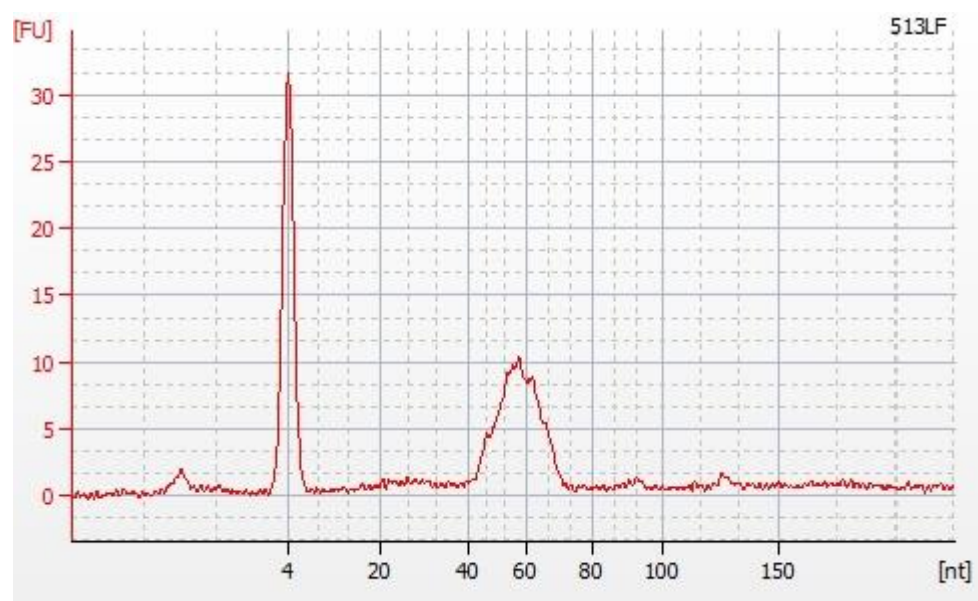

55

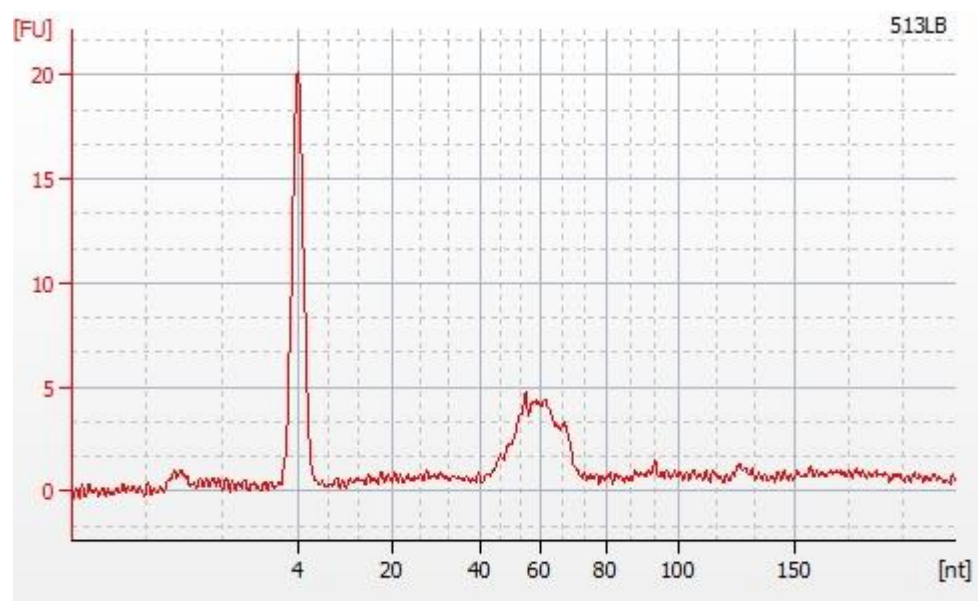

56

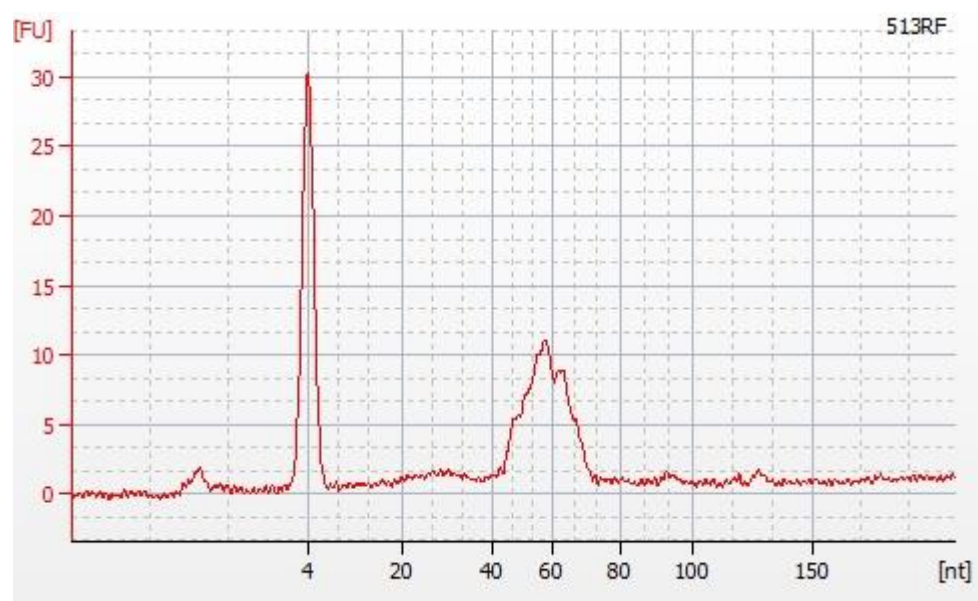

57

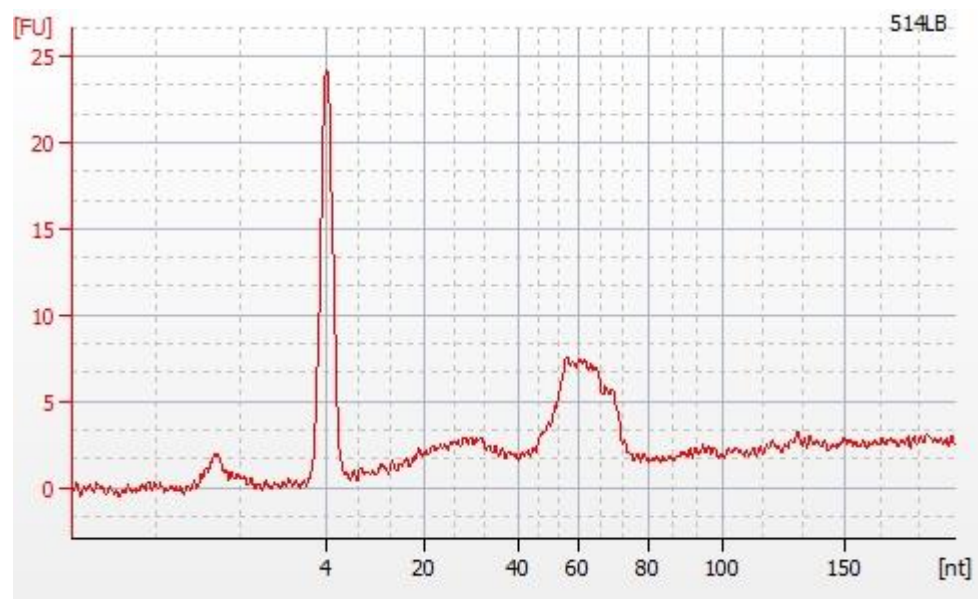

58

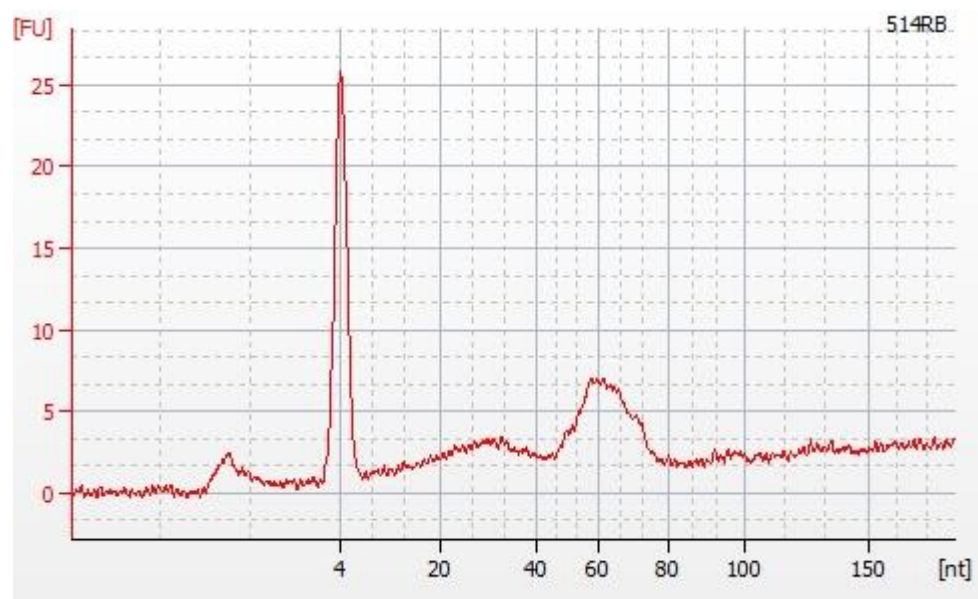

59

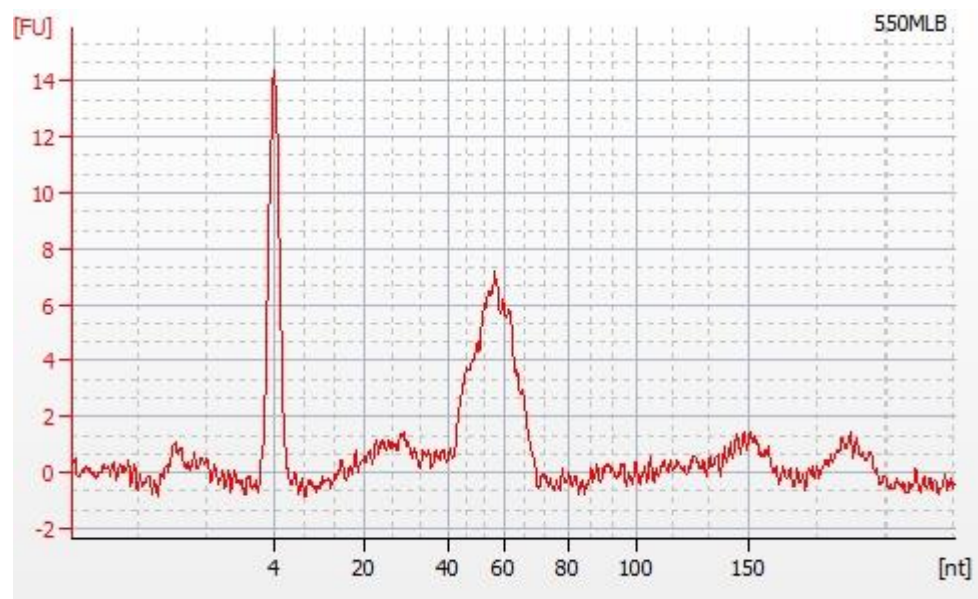

60

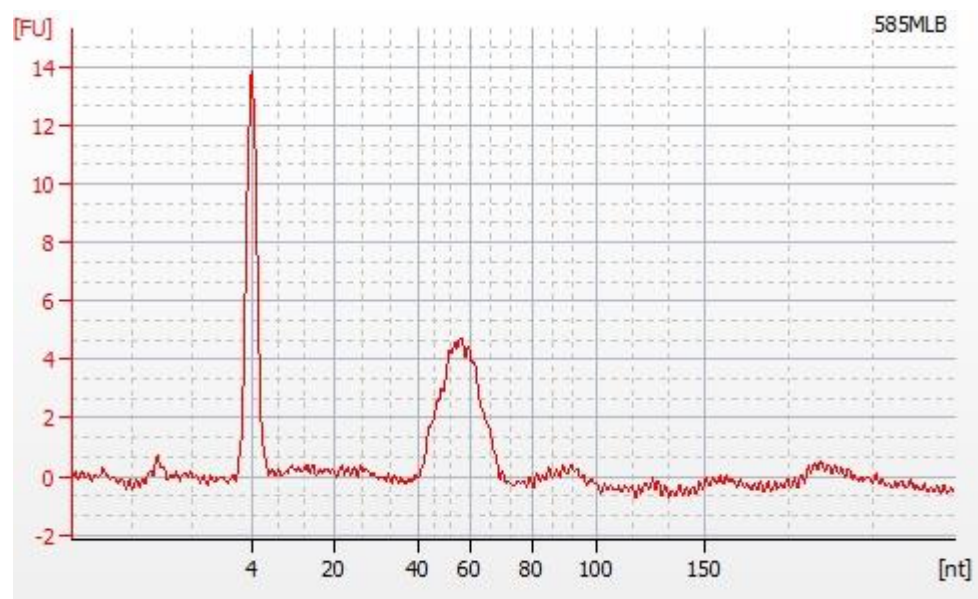

61

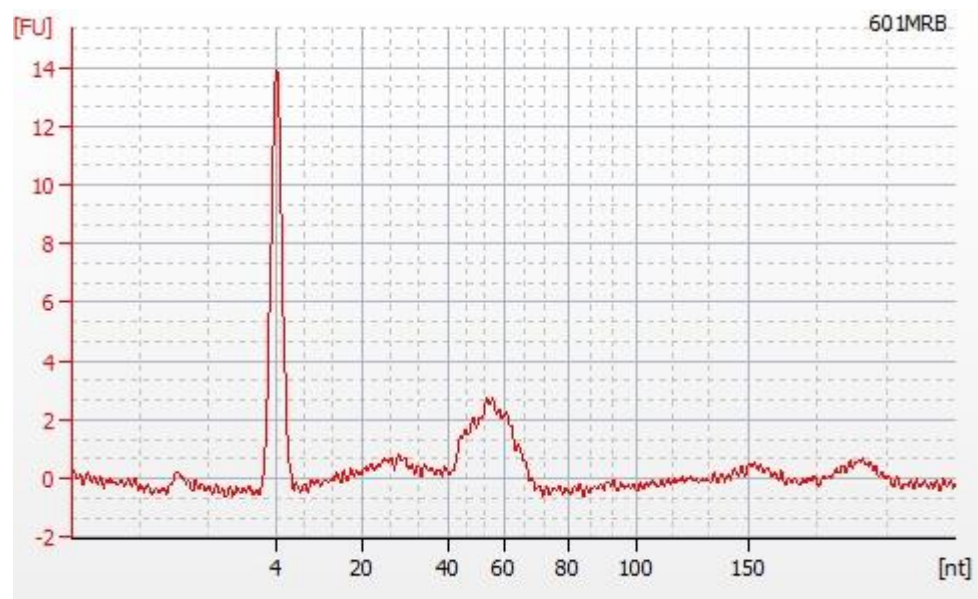

62

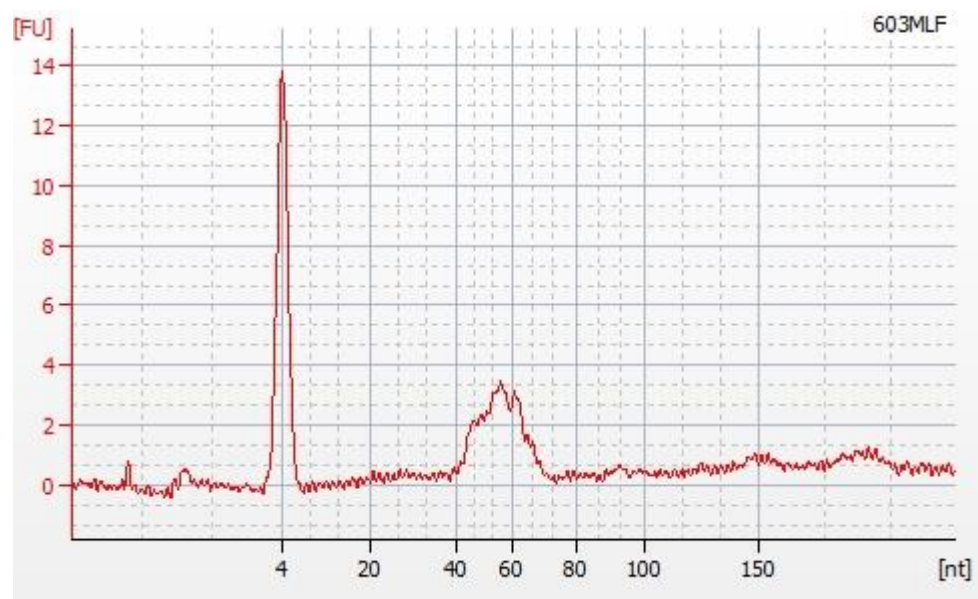

63

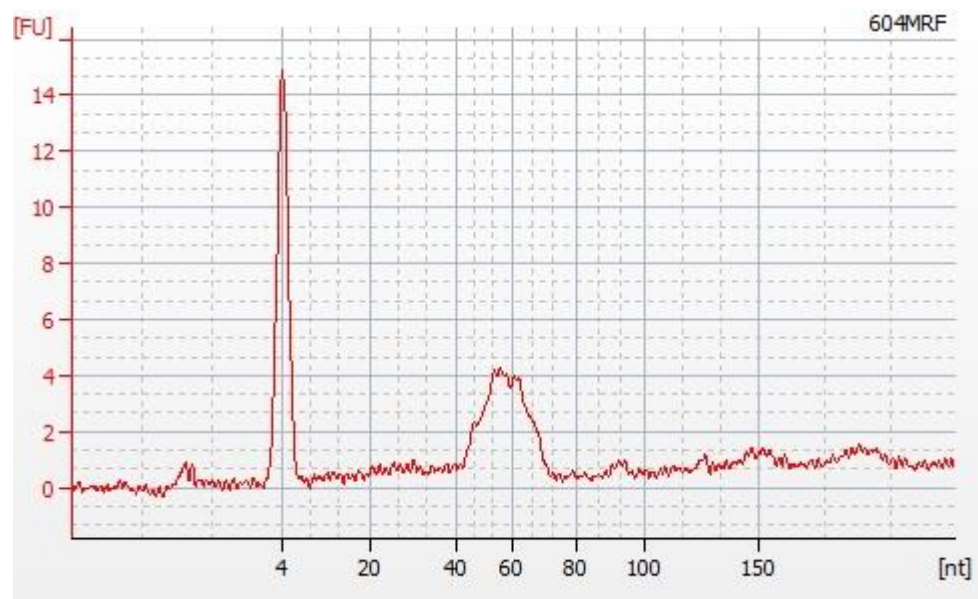

64

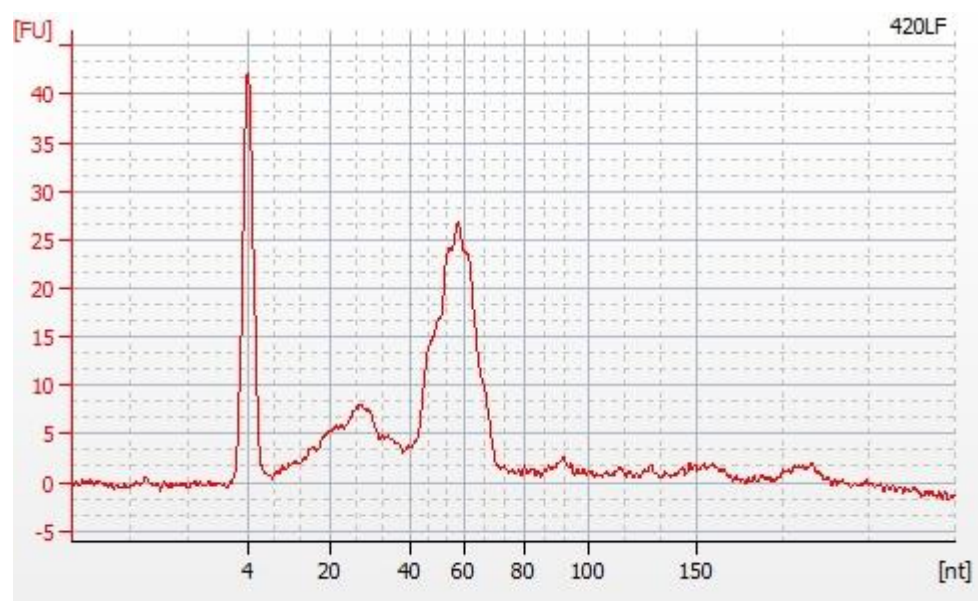

65

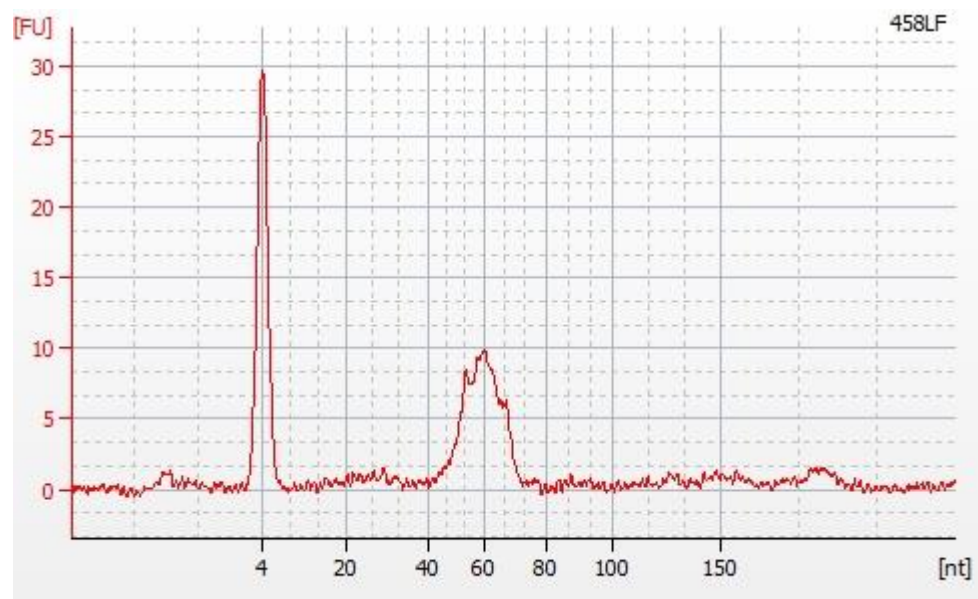

66

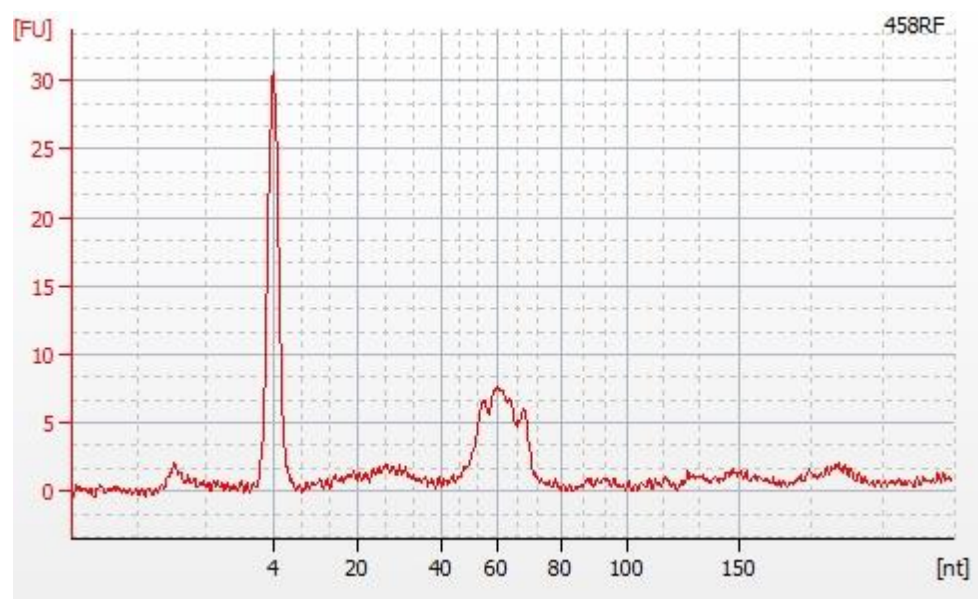

67

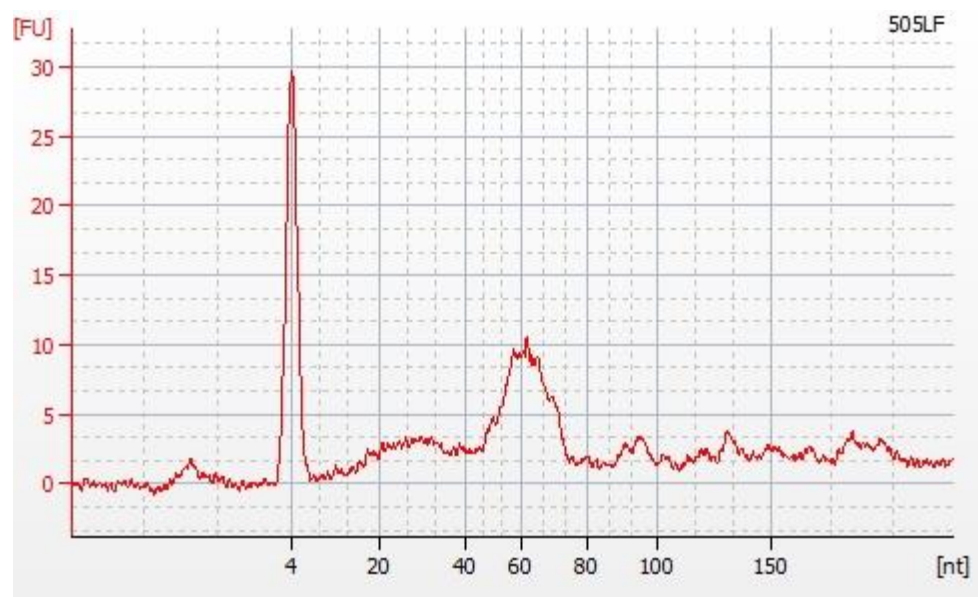

68

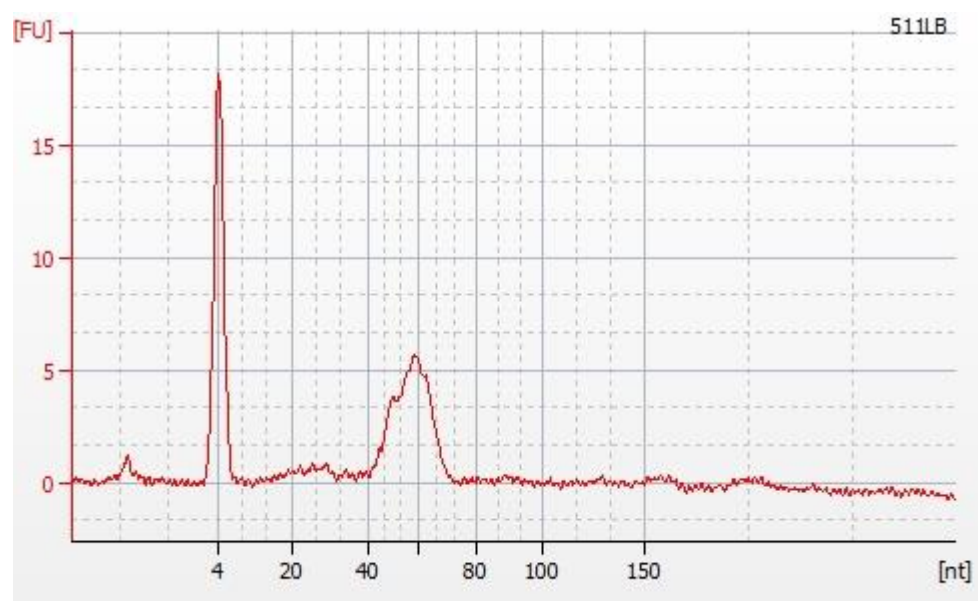

69

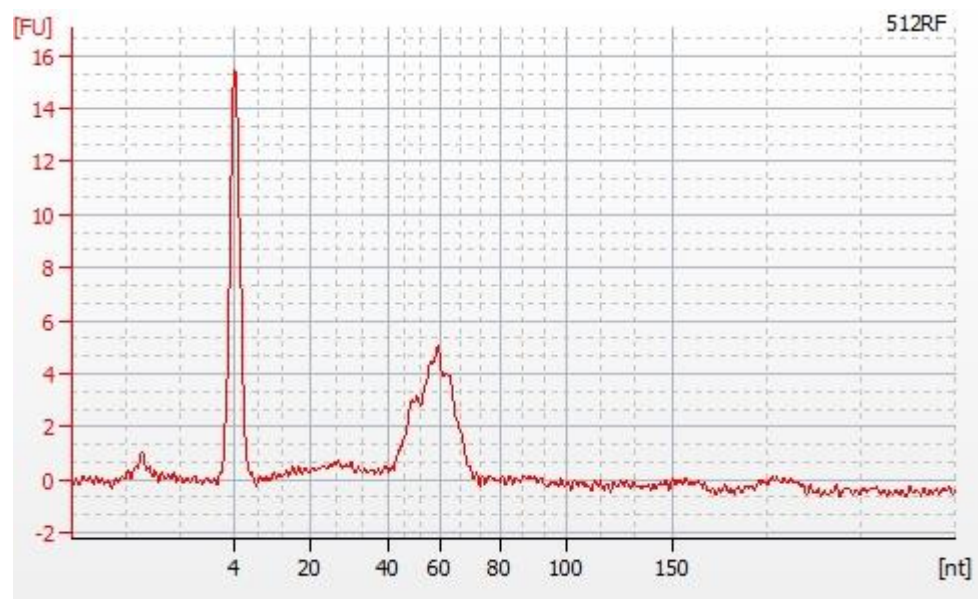

70

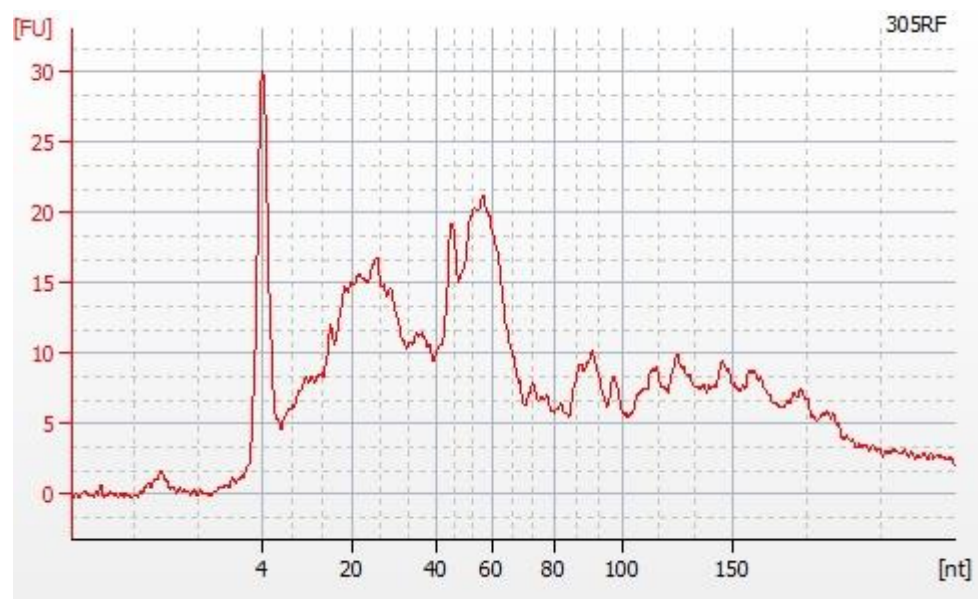

71

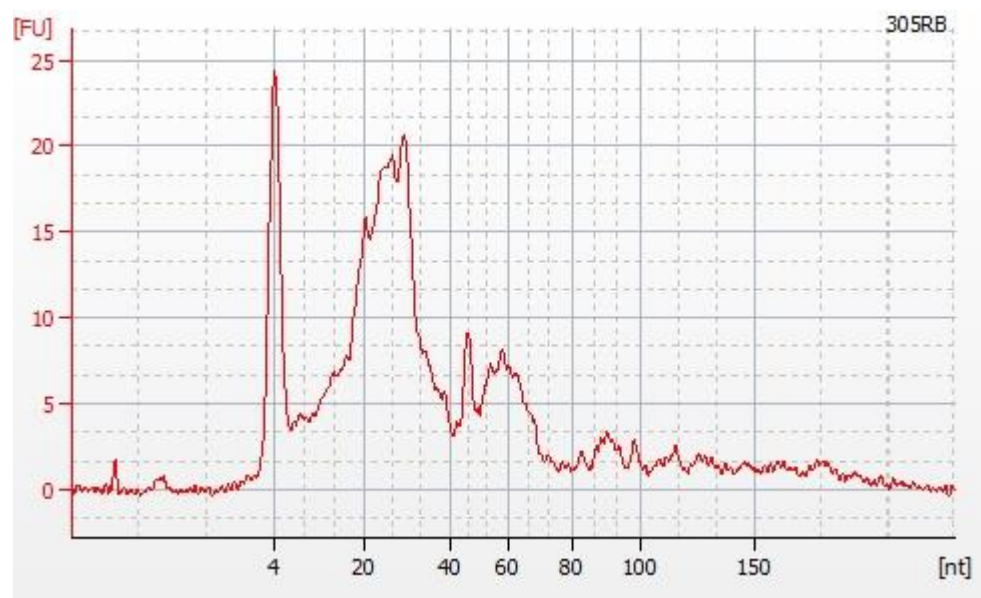

72

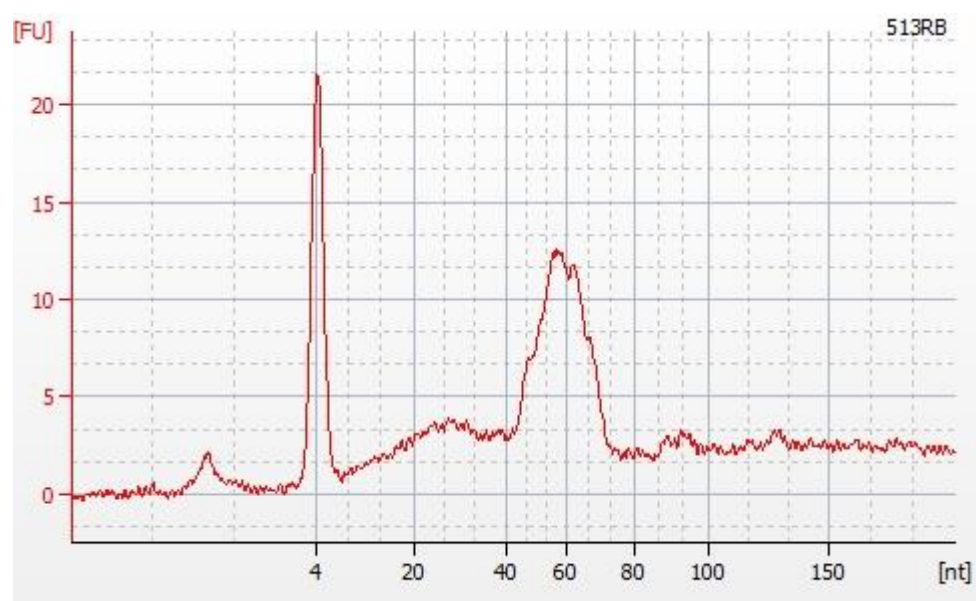

73

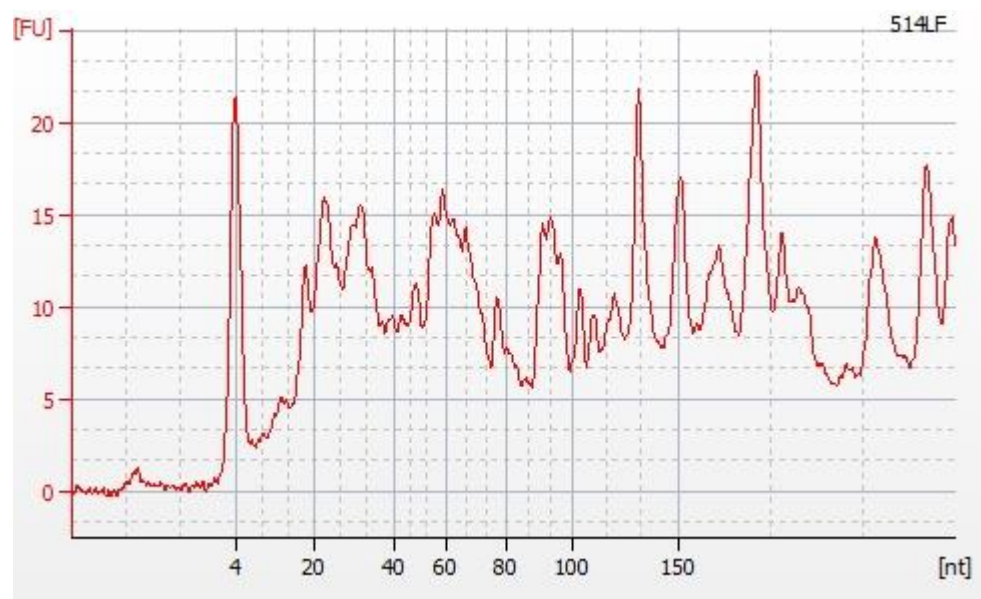

74

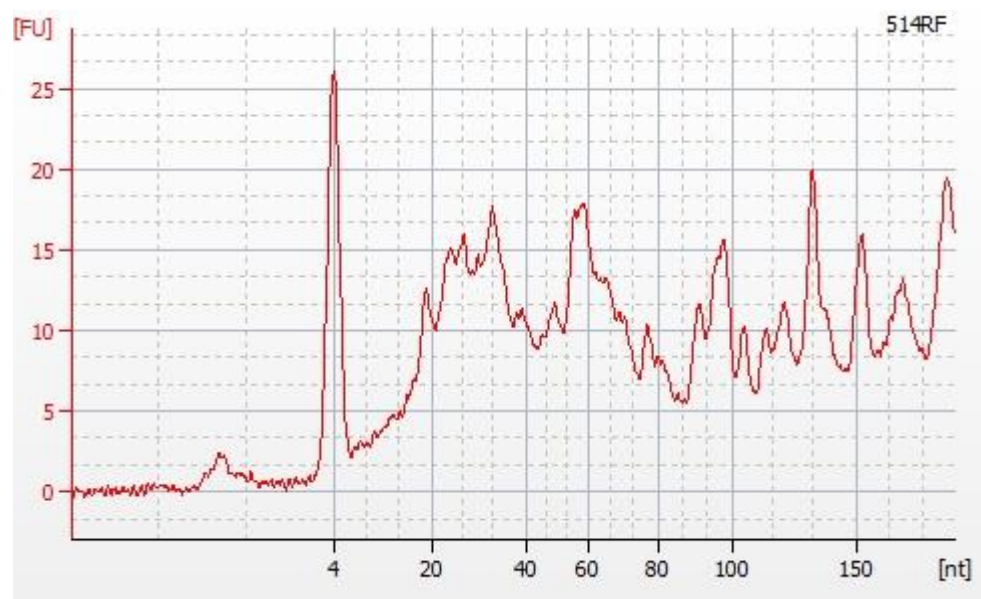

75

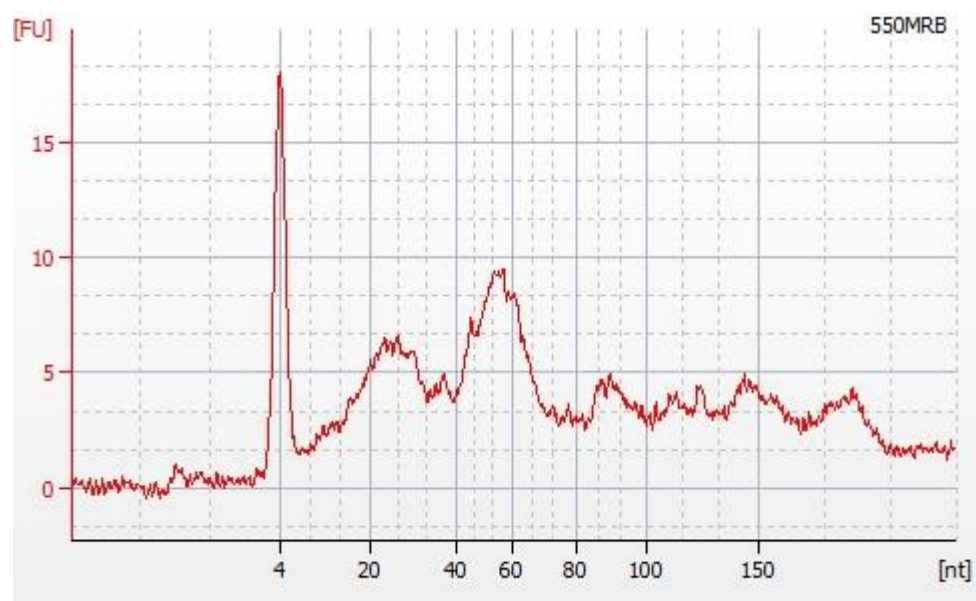

76

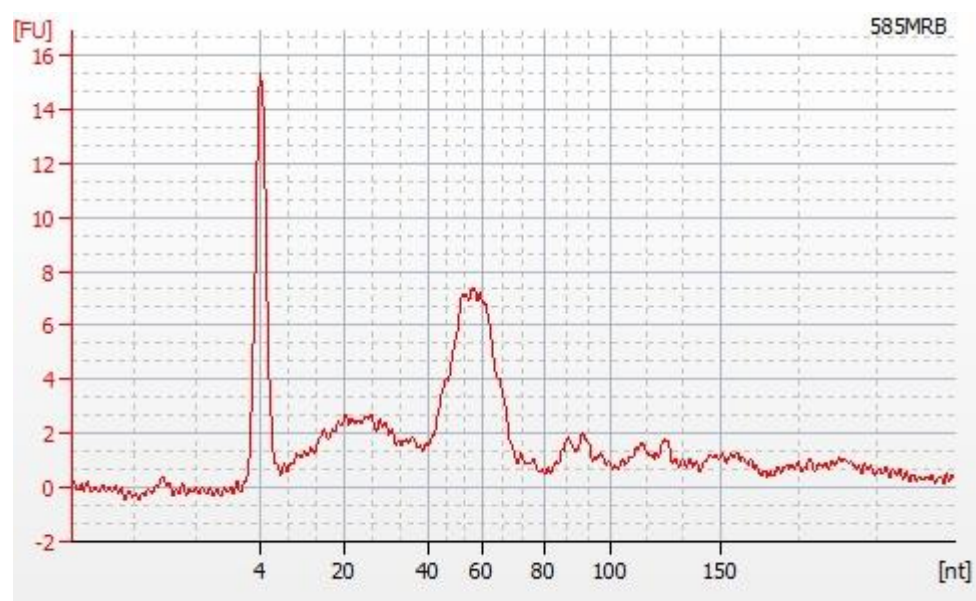

77

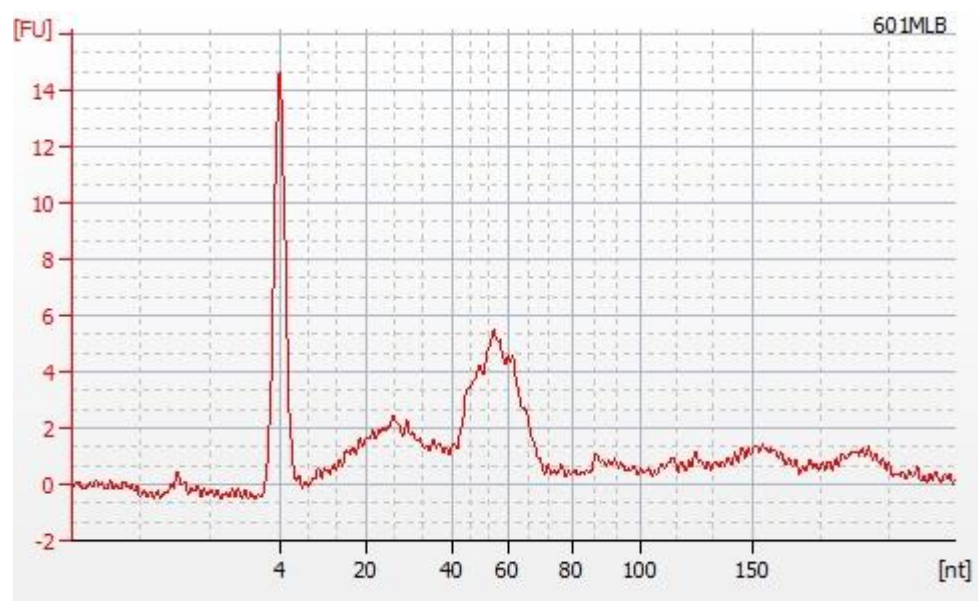

78

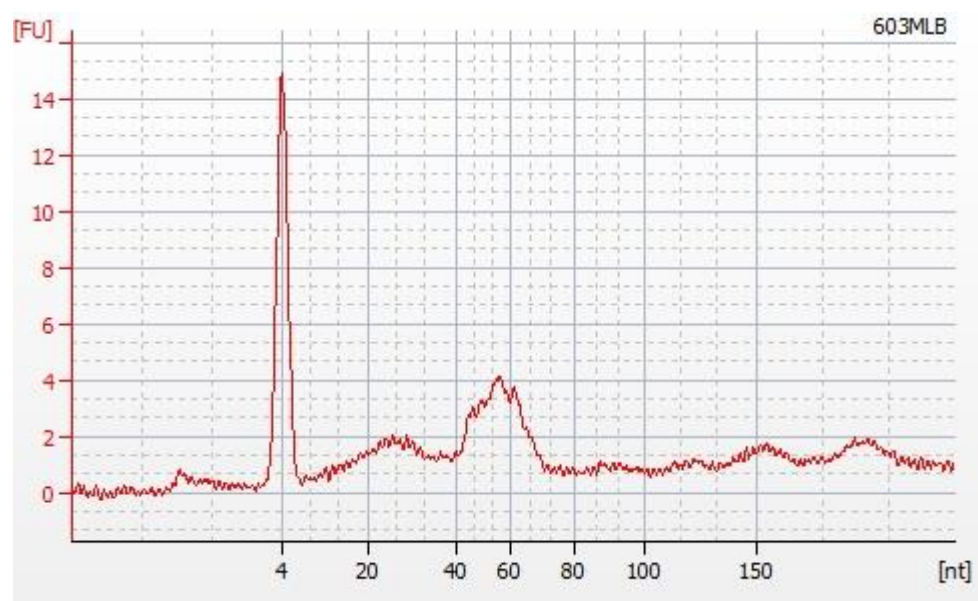

79

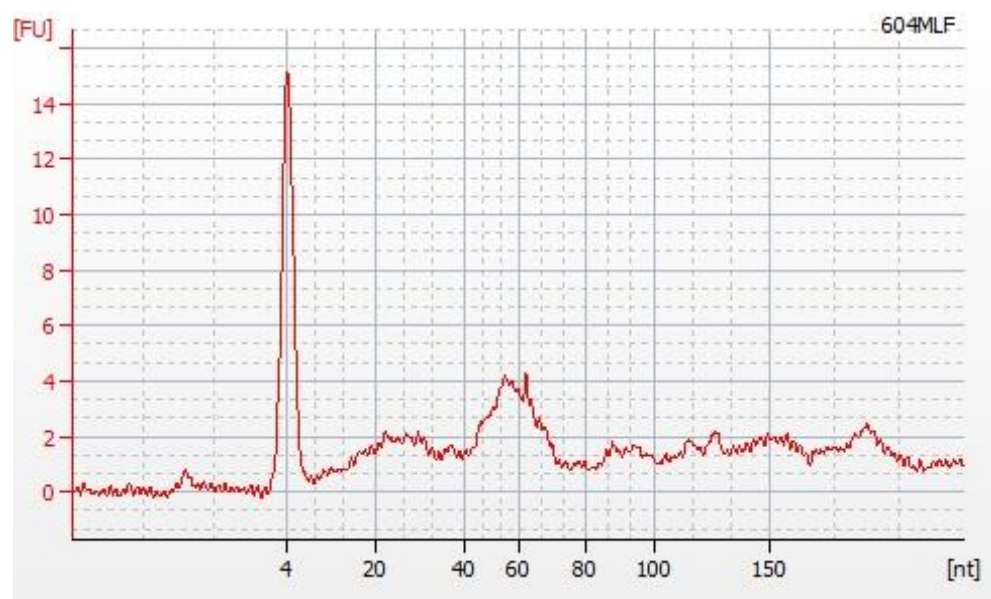

80

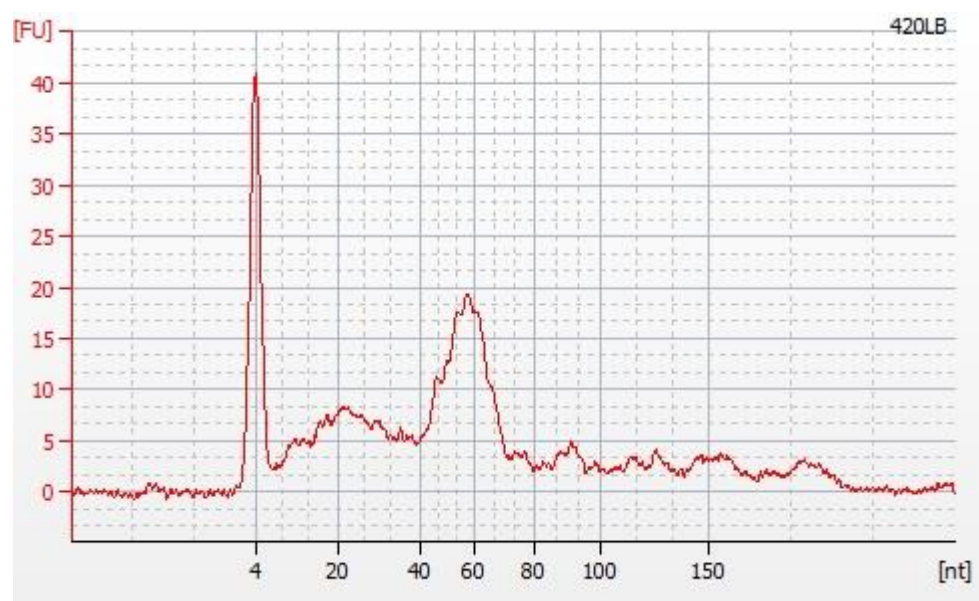

81

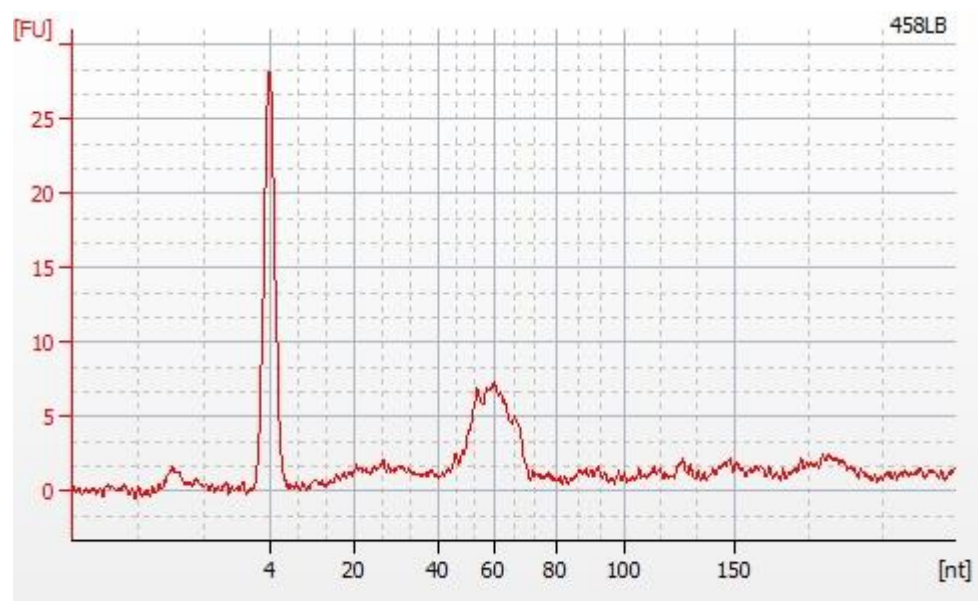

82

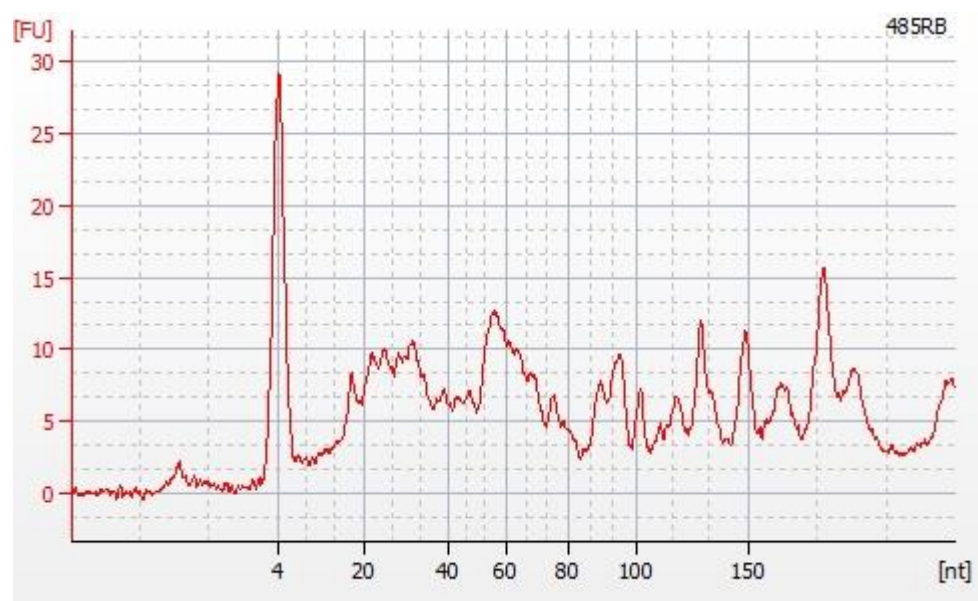

83

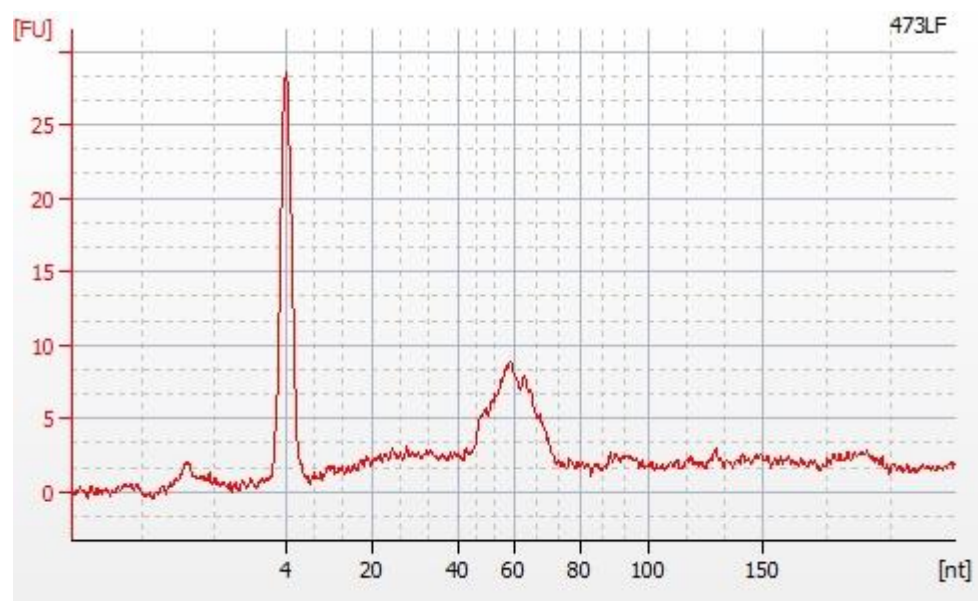

84

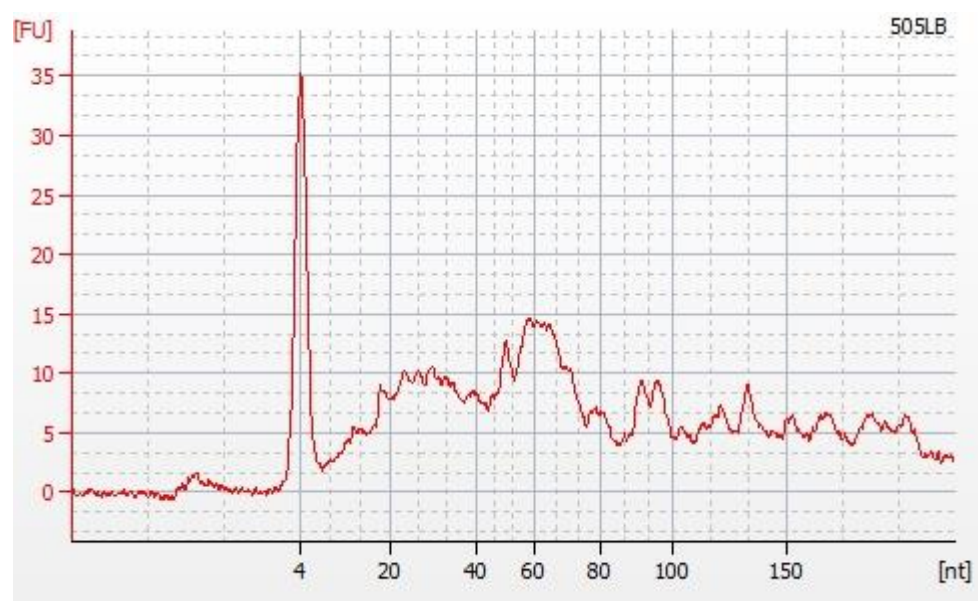

85

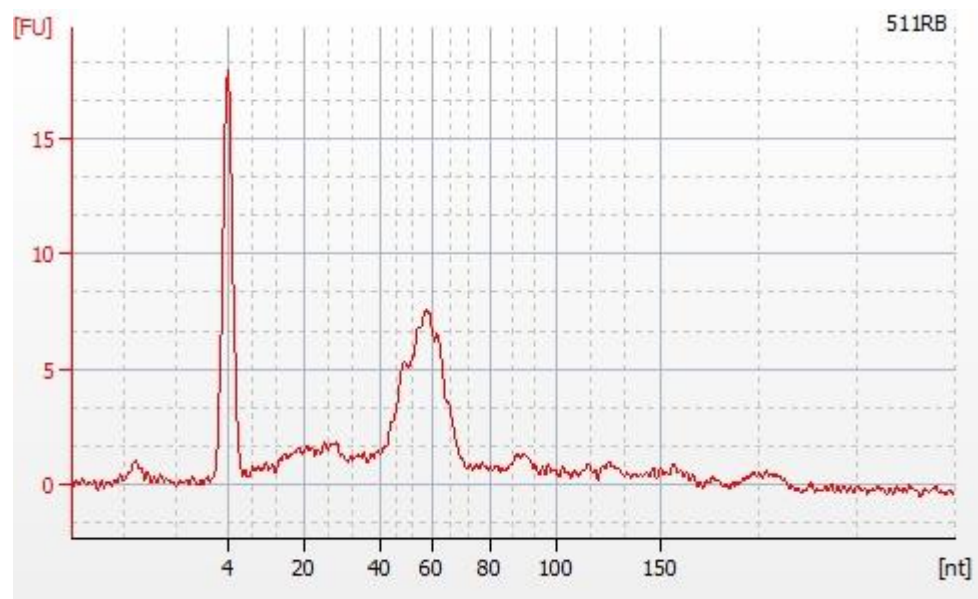

86

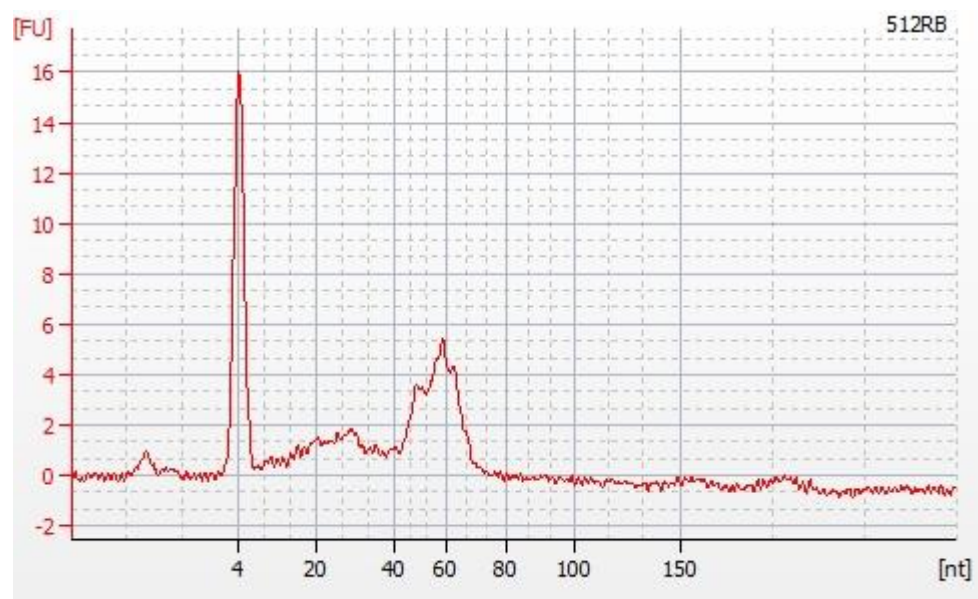

Supplement: S1 Fig — The numbers indicate sample number. (PDF) [file pone.0177182.s005.pdf]
